# Safety and antiviral activity of inhaled siRNA SNS812 for treatment of mild-to-moderate COVID-19: a phase 2 randomized trial

**DOI:** 10.1186/s12929-026-01284-5

**Published:** 2026-08-14

**Authors:** Shey-Ying Chen, Sui-Yuan Chang, Yi-Chung Chang, Ming-Che Liu, Kuan-Yuan Chen, Jer-Hwa Chang, Wen-Pin Tseng, Chien-Hao Lin, Jhong-Lin Wu, Pai-Chien Chou, Tsong-Yih Ou, Chin-Wang Hsu, Feng-Yi Chou, Hui-Ju Ho, Jen-Fu Yang, Chi-Fan Yang, Yi-Fen Chen, Kang-Yun Lee, William Lu, Pan-Chyr Yang

**Affiliations:** 1 Department of Emergency Medicine, College of Medicine, National Taiwan University Hospital, National Taiwan University, Taipei, Taiwan; 2 Department of Clinical Laboratory Sciences and Medical Biotechnology, National Taiwan University College of Medicine, Taipei, Taiwan; 3 Oneness Biotech Company, Limited, Taipei, Taiwan; 4 Department of Urology, Taipei Medical University Hospital, Taipei, Taiwan; 5 TMU Research Center of Urology and Kidney, Taipei Medical University, Taipei, Taiwan; 6 Clinical Research Center, Taipei Medical University Hospital, Taipei, Taiwan; 7 School of Dental Technology, College of Oral Medicine, Taipei Medical University, Taipei, Taiwan; 8 Division of Pulmonary Medicine, Department of Internal Medicine, Shuang Ho Hospital, Taipei Medical University, New Taipei, Taiwan; 9 School of Respiratory Therapy, College of Medicine, Taipei Medical University, Taipei, Taiwan; 10 Division of Pulmonary Medicine, Department of Internal Medicine, Wan Fang Hospital, Taipei Medical University, Taipei, Taiwan; 11 Division of Pulmonary Medicine, Department of Internal Medicine, Taipei Medical University Hospital, Taipei, Taiwan; 12 Division of Thoracic Medicine, Department of Internal Medicine, School of Medicine, College of Medicine, Taipei Medical University, Taipei, Taiwan; 13 Division of Infectious Diseases, Department of Internal Medicine, Wan Fang Hospital, Taipei Medical University, Taipei, Taiwan; 14 Department of Emergency, School of Medicine, College of Medicine, Taipei Medical University, Taipei, Taiwan; 15 Emergency Department, Department of Emergency and Critical Medicine, Wan Fang Hospital, Taipei Medical University, Taipei, Taiwan; 16 Microbio (Shanghai) Biotech Company, Shanghai, China; 17 Division of Pulmonary Medicine, Department of Internal Medicine, School of Medicine, College of Medicine, Taipei Medical University, Taipei, Taiwan; 18 Department of Internal Medicine, National Taiwan University College of Medicine, No. 7 Chung-Shan South Road, Taipei, 100 Taiwan

**Keywords:** Antiviral agent, COVID-19, Severe acute respiratory syndrome coronavirus 2, Aerosol delivery, SiRNA

## Abstract

**Background:**

SARS-CoV-2 remains a global health threat because ongoing viral evolution and immune evasion reduce the effectiveness of existing therapies. SNS812 is an inhaled small interfering RNA targeting a highly conserved region of the viral RNA-dependent RNA polymerase gene, representing a strategy that may enable broader antiviral activity against emerging variants.

**Methods:**

In this phase 2, double-blind, randomised, placebo-controlled trial, adults with mild-to-moderate COVID-19 within 3 days of symptom onset were randomly assigned (1:1:1) to receive placebo or inhaled SNS812 (100 mg or 200 mg) once daily for 7 days (ClinicalTrials.gov identifier: NCT05941793). Safety was the primary endpoint. Secondary endpoints included the time to sustained alleviation (TTSA) and resolution (TTSR) for prespecified composite target symptoms and individual symptoms. Virological outcomes were exploratory.

**Results:**

A total of 135 participants were enrolled, with more than 90% infected with immune-evasive SARS-CoV-2 variants. No treatment-related adverse events or serious adverse events were reported. In exploratory analyses, the 200 mg SNS812 group showed a shorter median time to SARS-CoV-2 antigen negativity (2.9 vs 3.6 days; p = 0.007) and a faster viral load reduction rate (− 0.755 vs − 0.652; p = 0.040), demonstrating dose-dependent virological effects. In the modified intention-to-treat population, exploratory symptom analyses showed shorter TTSA and TTSR for prespecified target symptoms with SNS812 200 mg compared with placebo (median TTSR 6.1 vs 8.1 days; adjusted hazard ratio 2.07, 95% CI 1.30–3.29; median TTSA 3.6 vs 6.5 days; adjusted hazard ratio 1.81, 95% CI 1.14–2.88).

**Conclusion:**

Inhaled SNS812 was safe and well tolerated and showed dose-dependent antiviral activity, with exploratory signals of symptomatic improvement in adults with mild-to-moderate COVID-19 infected with immune-evasive variants. These findings support further evaluation in larger, adequately powered trials, including older and higher-risk populations.

**Supplementary Information:**

The online version contains supplementary material available at 10.1186/s12929-026-01284-5.

## Introduction

The rapid and continuous evolution of severe acute respiratory syndrome coronavirus 2 (SARS-CoV-2) remains a persistent public health concern. Highly mutated variants such as JN.1, KP.2, and KP.3 have repeatedly reshaped viral transmission dynamics [1, 2], while recently emerging lineages including NB.1.8.1 and XFG further demonstrate increased antigenic divergence and immune escape [3]. Serum from individuals previously infected with the XBB.1.5 variant showed a 5.6-fold reduction in neutralising activity against JN.1 after 6 months [4]. This underscores an important public health challenge, as the pace of vaccine development increasingly struggles to match the rapid evolution of the virus, thereby leaving vaccinated individuals vulnerable to reinfection, particularly with immune-evasive variants.

Current therapeutic approaches for COVID-19 consist mainly of nucleoside analogues, such as molnupiravir and remdesivir, and protease inhibitors, including the combination of nirmatrelvir and ritonavir. These agents mitigate viral replication; however, significant adverse effects and drug–drug interactions restrict their overall clinical utility [5, 6]. Notably, a recent study reported that the nirmatrelvir–ritonavir combination fails to improve symptoms or reduce disease severity in vaccinated individuals [7]. These limitations underscore the need for a safe, broadly applicable antiviral therapy that can be used across diverse patient populations and remains effective against immune-evasive variants.

Short-interfering RNA (siRNA) represents a novel class of nucleic-acid-based therapeutics that combines precision with a favourable safety profile. By targeting highly conserved regions of viral genomes, siRNAs are able to achieve broad-spectrum inhibition across diverse viral variants. Preclinical studies have demonstrated siRNA’s potential in combating SARS-CoV-2, supporting its development as a promising antiviral modality [8, 9]. Two inhaled siRNA therapeutics, ALN-RSV01 for respiratory syncytial virus and siR-7-EM/KK-46 for COVID-19, have progressed to clinical evaluation. Both agents demonstrated limited efficacy at tolerable doses due to toxicity-related constraints. ALN-RSV01, the first aerosolised siRNA, employed unmodified backbones that elicited immune stimulation, thereby restricting feasible dosing [10]. Although siR-7-EM/KK-46 incorporated locked nucleic acid (LNA) modifications to enhance stability, it did not achieve clinically meaningful therapeutic effects [11].

SNS812 (previously C6G25S), a novel siRNA therapeutic, inhibits viral replication by targeting a highly conserved region within the RNA-dependent RNA polymerase gene, conferring potential activity against diverse SARS-CoV-2 variants. Chemical modification with 2′-O-methyl and 2′-fluoro groups reduces immunogenicity and improves molecular stability. In preclinical studies, Chang et al. demonstrated that SNS812 showed a favourable safety profile and significant preventive and therapeutic efficacy in SARS-CoV-2–infected hACE2 transgenic mice [12].

A randomised, double-blind, placebo-controlled phase 1 clinical study conducted in the United States in 2023 demonstrated that inhaled SNS812 was safe and well tolerated across single (0.3–1.2 mg/kg) and multiple (0.6 and 1.2 mg/kg for 7 days) dosing regimens, with no serious or drug-related adverse events (AEs). Pharmacokinetic analyses showed rapid absorption (median Tmax 1.5–2 h), a half-life of 5–7 h, and an absence of antidrug antibody formation [13]. On the basis of these favourable safety and pharmacokinetic findings, we conducted a multicentre, randomised, double-blind, placebo-controlled phase 2 clinical trial to evaluate the safety, virological and preliminary symptom-based effects of the inhaled small interfering RNA therapeutic SNS812 in adults with mild-to-moderate COVID-19.

## Methods

### Trial design, participants, and oversight

This phase 2, randomised, placebo-controlled, double-blind trial was conducted from 19 September 2023 to 12 August 2024 at five centres in Taiwan and the United States. The trial was registered at ClinicalTrials.gov (NCT05941793) on July 12, 2023.

Eligible participants were adults aged ≥ 18 years with a body mass index of 18.0–32.0 kg/m2, laboratory-confirmed SARS-CoV-2 infection by RT-PCR within 3 days before treatment initiation, and onset of COVID-19–attributable symptoms within 3 days before day 1 dosing. Participants were required to have at least one prespecified COVID-19 symptom at baseline, to be current non-smokers with no smoking or nicotine-product use within the previous 3 months, and to have mild or moderate COVID-19. COVID-19 severity was classified according to U.S. Food and Drug Administration definitions (Table S1) [14]. Key exclusion criteria included severe or critical COVID-19, investigator-assessed need for hospitalisation or high risk of progression before randomisation, oxygen saturation ≤ 93% on room air, PaO₂/FiO₂ < 300 mmHg, respiratory rate ≥ 30 breaths per minute, or heart rate ≥ 125 beats per minute. Participants were also excluded if they had SARS-CoV-2 infection within the previous 3 months; recent treatment with SARS-CoV-2 monoclonal antibodies, COVID-19 antivirals, systemic or inhaled corticosteroids for COVID-19, investigational drugs, COVID-19 or non-COVID-19 vaccines, human COVID-19 immunoglobulin, or convalescent plasma within protocol-defined windows; clinically relevant nasopharyngeal abnormalities or recent rhinitis that could interfere with assessments; clinically significant systemic diseases or active infections other than COVID-19; known HCV, HIV, or active HBV infection; moderate or severe hepatic or renal disease; clinically significant laboratory abnormalities; inability to use a nebuliser with a face mask; pregnancy or breastfeeding; or any other condition that, in the investigator’s judgment, could affect informed consent, protocol compliance, interpretation of study results, or participant safety. Detailed inclusion and exclusion criteria are provided in the Supplementary Appendix.

The trial adhered to the principles outlined in the Declaration of Helsinki, the International Council for Harmonisation of Technical Requirements for Pharmaceuticals for Human Use–Good Clinical Practice guidelines, and the CONSORT criteria for randomised trials. Ethics committee approval was obtained at each centre, and all participants provided written informed consent. Safety was monitored by the sponsor (Oneness Biotech Co., Ltd.) and an independent Data and Safety Monitoring Board (Supplementary Appendix, Appendix 1), which conducted a cumulative safety review after 27 participants completed their day 14 visits or withdrew from the trial. Academic investigators and sponsor employees jointly developed the study protocol. Investigators ensured protocol adherence and data integrity throughout the trial. The sponsor participated in protocol development and trial coordination. To support the independence and integrity of data analysis, all statistical analyses were performed according to a prespecified statistical analysis plan by an independent third-party contract research organisation, Bestat Pharmaservices Corp., before manuscript finalisation. Academic investigators had access to the relevant trial data, contributed to data interpretation, and participated in manuscript drafting and revision. All authors reviewed and approved the final manuscript and agreed with the decision to submit for publication. No Confidential Disclosure Agreement was in place between the sponsor and the academic authors or their institutions.

### Recruitment, randomization, and blindness

Participants were randomised in a 1:1:1 ratio using an interactive web-based randomisation system to receive SNS812 administered by inhalation at doses of 200 mg or 100 mg, or matching placebo once daily for seven consecutive days. Participants and investigators were blinded to treatment allocation. SNS812 was prepared in normal saline and administered via a mesh nebuliser (Air Pro III, Feellife Health Inc.) in accordance with the protocol. The placebo was composed of normal saline and was visually indistinguishable from the active drug of the SNS812 groups. Aerosol performance of SNS812 was assessed prior to clinical use using standard cascade impaction methods, as described in the Supplementary Appendix (Appendix 2), with aerosol characterisation data provided in Table S2 (Appendix 3).

### End-points of the trial

The primary end-points were safety, assessed by the incidence and severity of treatment-emergent adverse events (TEAEs), treatment-related adverse events, and serious adverse events throughout the study period. Secondary end-points evaluated symptom-based effects, including the time to symptom alleviation (TTSA) and time to symptom resolution (TTSR) of nine prespecified targeted COVID-19 symptoms through day 28 (Table S3), as well as the pharmacokinetic parameters of SNS812. The nine targeted symptoms were selected from a broader set of 14 COVID-19-associated symptoms recorded in the participant symptom diary (Table S4), which were broadly aligned with common COVID-19-related symptoms described in FDA guidance for outpatient COVID-19 clinical trials. These nine symptoms—fever or feeling hot, sore or dry throat, chills or shivers, headache, shortness of breath or difficulty breathing, nausea, vomiting, loss of smell, and loss of taste—were selected for the composite TTSA and TTSR analyses based on their relevance to acute COVID-19 manifestations and the inhaled, locally delivered nature of SNS812.

TTSA was defined as the time from the first dose to the day on which a symptom that was moderate or severe at baseline became mild or absent, or a symptom that was mild or absent at baseline became absent, for three consecutive days. TTSR was defined as the time from the first dose to the day on which symptoms were absent for three consecutive days, as assessed using a four-point symptom severity scale (Table S4). Exploratory end-points included changes in SARS-CoV-2 viral load over time, time to SARS-CoV-2 antigen negativity, TTSA and TTSR of each of the 14 individual COVID-19-associated symptoms through day 28, immunogenicity, and cytokine responses.

### Steps and details of the trial

This trial consisted of a screening phase, a 7-day treatment phase, and a 53-day follow-up phase for each participant. Participants assessed the severity of 14 COVID-19-associated symptoms twice daily from treatment initiation (day 1) through day 14 and once daily from day 15 to day 28. Symptom severity was recorded using a four-point scale, with scores of 0, 1, 2, and 3 indicating no, mild, moderate, and severe symptoms, respectively, and documented in electronic diaries (Table S4). Laboratory tests, including hematologic and blood biochemical panels, bleeding time, coagulation profiles, and urinalysis, were conducted at screening and on days 2, 4, 7, 9, 14, 28, and 60. Blood samples for pharmacokinetics study were collected before dosing on day 1 and day 7, and 1 h and 2 h (± 30 min) after the start of day 7 dose to determine SNS812 plasma concentration. Blood samples for cytokines analysis, including CXCL5, G-CSF, and IL-6, were collected on day 1, 2, 4, and 7. Blood samples for immunogenicity study were collected on day 1, 28, and 60. Electrocardiography assessments were conducted at screening and on days 1, 7, 9, 14, and 28. Investigators continuously monitored and assessed AEs throughout the treatment and follow-up phases after the first dose. Nasopharyngeal swab specimens were collected during screening and on days 1, 5, 7, 9, 14, and 28 (once daily), as well as on days 2, 3, and 4 (twice daily), for quantifying SARS-CoV-2 RNA via the RT-PCR assay at a central laboratory. Trained study nurses collected specimens using COPAN Universal Transport Medium (UTM) 330 C (Copan Diagnostics Inc., Murrieta, CA) and FLOQSwabs 503CS01 (Copan Diagnostics Inc., Murrieta, CA), following standardized protocols. The SARS-CoV-2 antigen test detecting viral nucleocapsid protein (Elecsys^®^ SARS-CoV-2 Antigen, Roche Diagnostics) was performed in parallel with RT-PCR at each study site unless two consecutive rapid antigen tests yielded negative results, in which case further antigen testing was discontinued.

### Safety causality assessment

Safety assessments included TEAEs, serious adverse events, adverse events leading to treatment discontinuation or study withdrawal, laboratory abnormalities, vital signs, electrocardiograms, physical examinations, cytokine responses, and immunogenicity. Investigators assessed AE severity according to the fifth version of Common Terminology Criteria for Adverse Events (CTCAE) developed by the United States National Cancer Institute [15]. Causality between specific AE and trial drug was judged by investigators, following WHO-UMC causality assessment system, by considering temporal relationship to study treatment, biological plausibility, alternative explanations including underlying COVID-19 or pre-existing comorbid medical conditions, concomitant medications, clinical course after continued dosing or treatment completion, and resolution pattern. Laboratory abnormalities were reviewed in the context of baseline values, medical history, intercurrent illness, physical activity, and repeat testing. Events were considered related to the investigational product when a reasonable possibility of causal association could not be excluded.

### Statistical analysis

A Statistical Analysis Plan (SAP) was developed and approved prior to database lock and prespecified the statistical methods for safety and efficacy analyses. Sample size estimation was based on the change from baseline in viral load across three treatment arms. Therefore, although TTSA and TTSR were prespecified secondary endpoints, the study was designed primarily to evaluate safety and provide preliminary evidence of antiviral activity, and was not powered to establish definitive clinical benefit based on symptom-based outcomes. Assuming a mean reduction of − 2.4 in the treatment groups versus − 1.5 in the placebo group, with a common standard deviation of 1.3, 38 participants per group were required to achieve 80% power at a two-sided α of 0.05 using a Wilcoxon rank-sum test. Allowing for a 15% dropout rate, approximately 45 participants per group were planned. No interim efficacy analysis was planned. An independent Data and Safety Monitoring Board reviewed cumulative safety data up to day 14 after 27 participants had completed the day 14 visit. Statistical analysis populations are detailed in Table S5.

All statistical analyses were performed using SAS^®^ version 9.4 or later (SAS Institute Inc., Cary, NC). Continuous variables were summarised using the number of observations, mean, standard deviation, median, and range, while categorical variables were summarised using frequencies and percentages. Baseline demographic, viral genotype and primary end-points were summarised using descriptive statistics. Continuous variables were analysed using analysis of variance or the Kruskal–Wallis test, as appropriate, and categorical variables using Fisher’s exact test. Analyses of target symptoms and each individual symptom TTSR and TTSA were conducted using Kaplan–Meier methods and log-rank tests. Bonferroni correction for multiple comparisons among the three study groups was applied for each individual COVID-19 symptom. Virological analyses in the modified intention-to-treat population included Kaplan–Meier analyses of time to SARS-CoV-2 antigen negativity and analysis of covariance for viral load changes adjusted for baseline viral load. Pharmacokinetic and immunogenicity endpoints were analysed descriptively.

## Results

### Characteristics of included participants

**Fig. 1:**
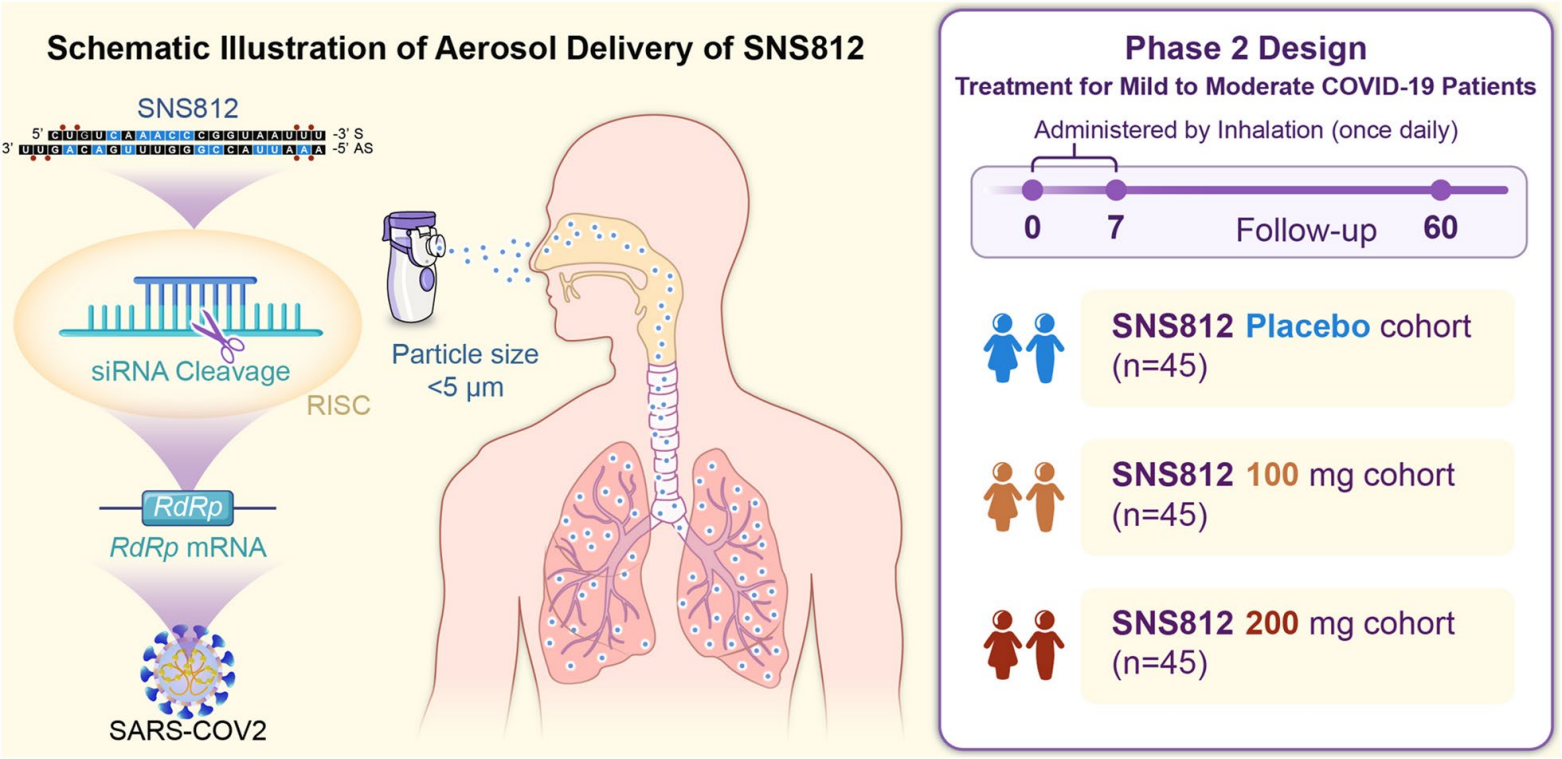
Overview of the study design and delivery of SNS812 is a fully chemically modified siRNA targeting a highly conserved region of the SARS-CoV-2 *RdRP* gene. The siRNA incorporates 2′-O-methyl modifications (black squares), 2′-fluoro modifications (blue squares), and phosphorothioate linkages (red dots) to enhance molecular stability and reduce immune stimulation. SNS812 is administered as an inhaled aerosol via a handheld mesh nebuliser. Following cellular uptake, SNS812 is incorporated into the RNA-induced silencing complex (RISC), leading to sequence-specific cleavage and suppression of viral *RdRP* mRNA. The clinical trial enrolled patients with mild-to-moderate COVID-19 within three days of symptom onset. A total of 135 participants were randomised in a 1:1:1 ratio to receive placebo, SNS812 100 mg, or SNS812 200 mg (45 participants per group). Study treatment was initiated on Day 0 and administered once daily by inhalation for seven consecutive days, followed by a 53-day post-treatment follow-up period

**Fig. 2:**
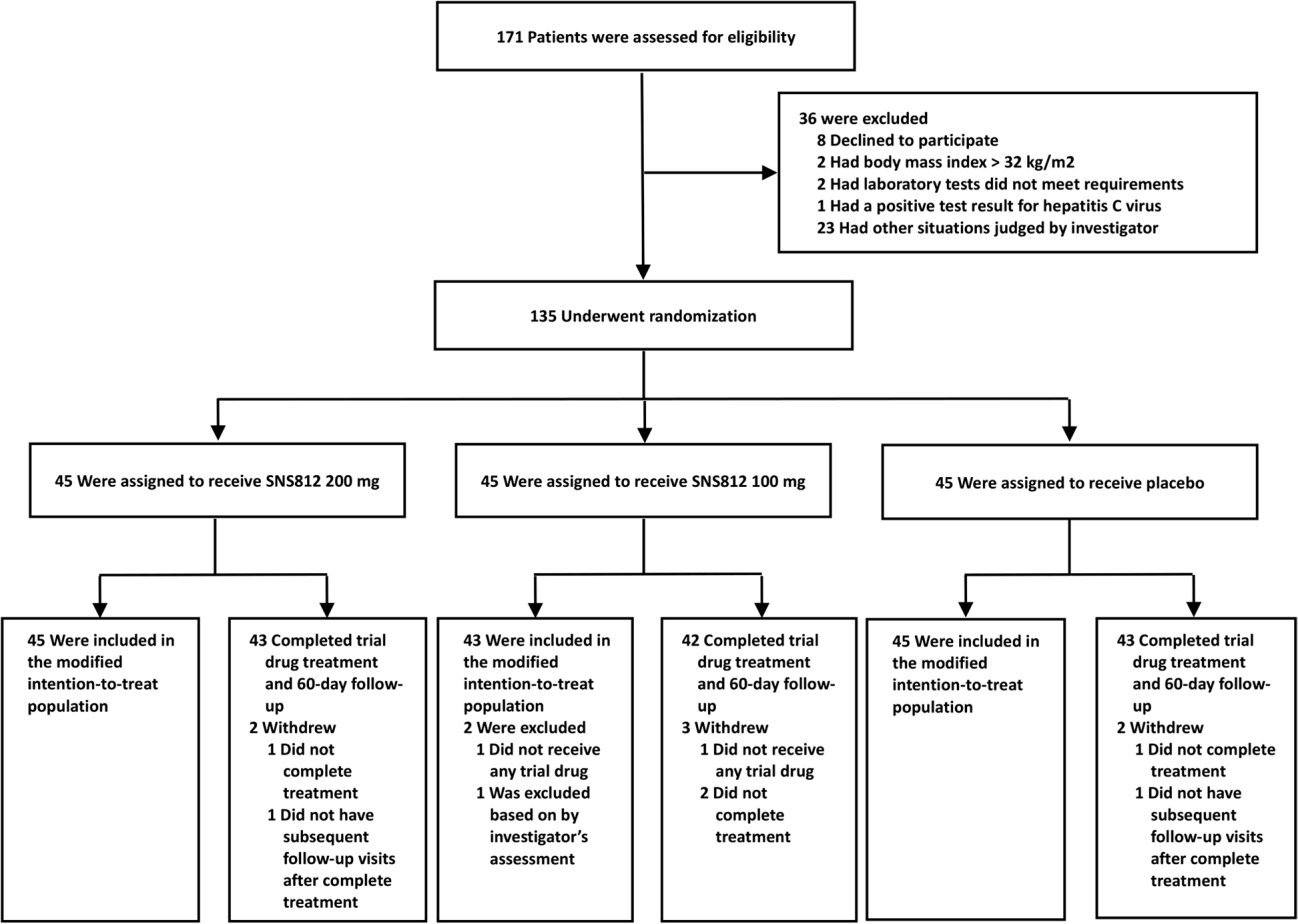
Definition of analysis populations and patient disposition. The mITT population comprised all randomized patients without any remarkable medical history may impact patient-reported outcomes, received at least one dose of the trial drug or placebo, performed baseline and at least had one post-baseline visit and SARS-CoV-2 antigen test evaluation. All randomized patients comprised the full analysis set for demographic baseline characteristics analyses. One patient did not receive any trial drug and was excluded from the safety set and mITT population. One another patient was excluded from the mITT population based on the investigator’s assessment that the patient’s preexisting chronic condition could significantly impact the efficacy evaluation

Participants were randomised to receive SNS812 200 mg, SNS812 100 mg, or placebo (n = 45 per group; full analysis set). An overview of the study design and participant disposition is shown in Figs. 1 and 2. Seven participants withdrew after randomisation, including one who did not receive study treatment and was excluded from the safety set. A total of 128 participants completed treatment and follow-up, with high treatment adherence across all groups (> 93%). Baseline characteristics were well balanced among the three groups (Table 1). The study population was relatively young (median age, 36 years) and predominantly female (63%), with no comorbidities reported in most participants. Overall, 43.0% had at least one risk factor for severe COVID-19, most commonly overweight or obesity and hypertension (Table S6). All participants had mild COVID-19 at baseline, had previously completed at least one full course of COVID-19 vaccination, and none were current smokers. All participants initiated treatment within 72 h of symptom onset, with 68.9% treated within 48 h. Baseline viral loads were comparable across groups. Viral genotyping by next-generation sequencing identified JN.1 as the predominant variant, followed by LB.1 and KP.2; detailed genotype distributions are provided in Table S7.

**Table 1:** demographic and clinical characteristics of the participants at baseline (full analysis set)

Characteristics	SNS812 200 mg (N = 45)	SNS812 100 mg (N = 45)	Placebo (N = 45)	Total (N = 135)
Median age (range) – yr	39 (19–67)	36 (23–62)	36 (24–66)	36 (19–67)
Male sex – no. (%)	20 (44.4)	17 (37.8)	13 (28.9)	50 (37.0)
Current smoker	0 (0.0)	0 (0.0)	0 (0.0)	0 (0.0)
Risk factors for severe Covid-19 – no. (%)				
At least one risk factor	21 (46.7)	20 (44.4)	17 (37.8)	58 (43.0)
Age ≧ 65 yr	1 (2.2)	0 (0.0)	2 (4.4)	3 (2.2)
Overweight or obesity*	18 (40.0)	15 (33.3)	15 (33.3)	48 (35.6)
Overweight	17 (37.8)	11 (24.4)	15 (33.3)	43 (31.9)
Obesity	1 (2.2)	4 (8.9)	0 (0.0)	5 (3.7)
Diabetes mellitus	1 (2.2)	1 (2.2)	1 (2.2)	3 (2.2)
Hypertension	5 (11.1)	4 (8.9)	5 (11.1)	14 (10.4)
Coronary artery disease	0 (0.0)	0 (0.0)	1 (2.2)	1 (0.7)
Chronic liver disease†	0 (0.0)	1 (2.2)	1 (2.2)	2 (1.5)
Asthma	0 (0.0)	2 (4.4)	1 (2.2)	3 (2.2)
Depression	1 (2.2)	0 (0.0)	0 (0.0)	1 (0.7)
Chronic kidney disease	2 (4.4)‡	0 (0.0)	0 (0.0)	2 (1.5)
Thalassemia	1 (2.2)	0 (0.0)	1 (2.2)	2 (1.5)
COVID-19 vaccination naive	0 (0.0)	0 (0.0)	0 (0.0)	0 (0.0)
Covid-19 severity in screening – no. (%)				
Mild	45 (100.0)	45 (100.0)	45 (100.0)	135 (100.0)
Moderate§	0 (0.0)	0 (0.0)	0 (0.0)	0 (0.0)
Median total score for the 14 symptoms at enrollment (range) ¶, ¥	12 (5–21)	12 (3–25)	12 (4–28)	12 (3–28)
Interval from symptom onset to initiation of trial drug or placebo				
< 24 h – no. (%)	0 (0.0)	3 (6.7)	0 (0.0)	3 (2.2)
24–48 h – no. (%)	32 (71.1)	28 (62.2)	30 (66.7)	90 (66.7)
48–72 h – no. (%)	13 (28.9)	14 (31.1)	15 (33.3)	42 (31.1)
Viral load—log_10_ copies per milliliter (mean ± SD)	8.49 ± 1.09	8.42 ± 0.87	8.53 ± 0.9	8.48 ± 0.95
SARS-CoV-2 strain genotype – no. (%)				
Omicron (BA.2-like)	42 (93.3)	43 (95.6)	44 (97.8)	129 (95.6)
JN.1 (%)	20 (44.4)	25 (55.6)	22 (48.9)	67 (49.6)
KP.1 (%)	4 (8.9)	1 (2.2)	3 (6.7)	8 (5.9)
KP.2 (%)	5 (11.1)	5 (11.1)	5 (11.1)	15 (11.1)
KP.3 (%)	1 (2.2)	3 (6.7)	3 (6.7)	7 (5.2)
KP.4 (%)	1 (2.2)	0 (0.0)	0 (0.0)	1 (0.7)
LB.1 (%)	9 (20.0)	5 (11.1)	9 (20.0)	23 (17.0)
Others (%)	2 (4.4)	4 (8.9)	2 (4.4)	8 (5.9)
Omicron (XBB-like) (%)	3 (6.7)	2 (4.4)	1 (2.2)	6 (4.4)

^*^ Overweight was defined as a body mass index (BMI, the weight in kilograms divided by the square of the height in meters) of 25 kg/m^2^ to 29 kg/m^2^, and obesity was defined as a BMI of ≥ 30 kg/m^2^ on the day of eligibility evaluation

^†^ Includes one case of non-viral chronic hepatitis and one case of hepatic steatosis

^‡^ One patient had a history of complement-mediated glomerulopathy and one patient had a history of idiopathic nephropathy; however, they both had normal renal function on enrollment

^§^ Patients were defined as having moderate COVID-19 if they did not meet the criteria for severe COVID-19, had an oxygen saturation of more than 93% while breathing room air, and met at least one of the following criteria: shortness of breath with activity or exercise, respiratory rate of 20 to 29 breaths per minute, or heart rate of 90 to 124 beats per minute

^¶^ Includes fever, cough, muscle or body aches, sore or dry throat, low energy or tiredness, chills or shivers, stuffy or runny nose, headache, shortness of breath or difficulty breathing, nausea, vomiting, diarrhea, loss of smell and loss of taste

^¥^ Patients assessed the severity of each of the 14 COVID-19-associated symptoms on a four-point scale from 0 to 3, with zero indicating no symptoms, 1 mild symptoms, 2 moderate symptoms, and 3 severe symptoms

### Safety endpoints

The incidence of TEAEs was similar across treatment groups from the first dose to day 60, with no participant discontinuing treatment because of AEs (Table 2). Most TEAEs were consistent with the natural course of COVID-19, and no clinically meaningful imbalances were observed across treatment groups or age subgroups (Tables S8 and S9). One participant in the 100-mg SNS812 group experienced a severe adverse event consisting of elevated alanine aminotransferase and aspartate aminotransferase levels. This participant had a history of idiopathic hepatitis with abnormal baseline liver enzymes; the elevations peaked on day 14 and resolved by day 28 without intervention, consistent with a self-limiting event. Transient increases in creatine phosphokinase were observed in the SNS812 groups and were attributed to vigorous physical activity during recovery (Figure S1). None of these participants had any known comorbid medical conditions. After review of timing, clinical context, alternative explanations, and resolution patterns, Investigators determined that no AEs were related to SNS812. No clinically significant abnormalities were identified in other safety assessments. Detailed safety data are provided in the Supplementary Appendix (Appendix 4).

**Table 2:** summary of adverse event (safety set)

	SNS812 200 mg	SNS812 100 mg	Pooled SNS812 Groups	Placebo
Events	(N = 45)	(N = 44)*	(N = 89)	(N = 45)
No. of events	50	57	107	51
Patients with an event – no. (%)				
Any adverse event	27 (60.0)	24 (54.6)	51 (57.3)	21 (46.7)
Adverse event considered by the investigator to be related to the trial drug or placebo	0 (0.0)	0 (0.0)	0 (0.0)	0 (0.0)
Severe adverse event	0 (0.0)	1 (2.3)†	1 (1.1)	0 (0.0)
Discontinued trial drug or placebo because of an adverse event	0 (0.0)	0 (0.0)	0 (0.0)	0 (0.0)
Withdrew from the trial because of an adverse event	0 (0.0)	0 (0.0)	0 (0.0)	0 (0.0)
Death during either treatment or the follow-up period	0 (0.0)	0 (0.0)	0 (0.0)	0 (0.0)
AEs during laboratory investigations				
Creatine phosphokinase level increase	5 (11.1)	2 (4.5)	7 (7.9)‡	0 (0.0)
White blood cell count decrease	3 (6.7)	1 (2.3)	4 (4.5)	2 (4.4)
Neutrophil count decrease	0 (0.0)	2 (4.5)	2 (2.2)	2 (4.4)
Alanine aminotransferase level increase	0 (0.0)	2 (4.5)	2 (2.2)	0 (0.0)
Aspartate aminotransferase level increase	0 (0.0)	2 (4.5)	2 (2.2)	0 (0.0)
Total bilirubin level increase	1 (2.2)	0 (0.0)	1 (1.1)	1 (2.2)
Creatinine level increase	1 (2.2)	0 (0.0)	1 (1.1)	1 (2.2)
Platelet count decrease	1 (2.2)	0 (0.0)	1 (1.1)	0 (0.0)
AEs from organ systems				
Constitutional				
Pyrexia	1 (2.2)	1 (2.3)	2 (2.2)	0 (0.0)
Chills	0 (0.0)	1 (2.3)	1 (1.1)	0 (0.0)
Myalgia	0 (0.0)	1 (2.3)	1 (1.1)	1 (2.2)
Malaise	0 (0.0)	1 (2.3)	1 (1.1)	2 (4.4)
Nervous system				
Headache	3 (6.7)	4 (9.1)	7 (7.9)	1 (2.2)
Dizziness	3 (6.7)	1 (2.3)	4 (4.5)	3 (6.7)
Loss of smell	3 (6.7)	7 (15.9)	10 (11.2)	10 (22.2)
Loss of taste	4 (8.9)	7 (15.9)	11 (12.4)	8 (17.8)
Respiratory tract, upper and lower				
Nasal congestion	3 (6.7)	2 (4.5)	5 (5.6)	1 (2.2)
Rhinorrhea	1 (2.2)	0 (0.0)	1 (1.1)	2 (4.4)
Throat pain	2 (4.4)	1 (2.3)	3 (3.4)	1 (2.2)
Cough	1 (2.2)	0 (0.0)	1 (1.1)	0 (0.0)
Dyspnea	1 (2.2)	0 (0.0)	1 (1.1)	2 (4.4)
Cardiac				
Palpitation	0 (0.0)	1 (2.3)	1 (1.1)	0 (0.0)
Gastrointestinal				
Nausea	1 (2.2)	3 (6.8)	4 (4.5)	0 (0.0)
Vomiting	0 (0.0)	2 (4.5)	2 (2.2)	1 (2.2)
Abdominal pain	1 (2.2)	0 (0.0)	1 (1.1)	0 (0.0)
Diarrhea	2 (4.4)	5 (11.4)	7 (7.9)	4 (8.9)
Musculoskeletal and soft tissue				
Back pain	1 (2.2)	0 (0.0)	1 (1.1)	0 (0.0)
Epicondylitis	1 (2.2)	0 (0.0)	1 (1.1)	0 (0.0)
Dermatological				
Rash	0 (0.0)	0 (0.0)	0 (0.0)	1 (2.2)
Purpura	0 (0.0)	0 (0.0)	0 (0.0)	1 (2.2)
Urticaria	0 (0.0)	1 (2.3)	1 (1.1)	0 (0.0)
Infections				
Upper respiratory tract, unspecified	2 (4.4)	1 (2.3)	3 (3.4)§	2 (4.4)‡
Acute sinusitis	1 (2.2)	0 (0.0)	1 (1.1)	1 (2.2)
Tonsillitis	0 (0.0)	1 (2.3)	1 (1.1)	0 (0.0)
Bronchitis	0 (0.0)	0 (0.0)	0 (0.0)	1 (2.2)
Neoplasm, benign, malignant, and unspecified				
Fibroadenoma of breast	1 (2.2)¶	0 (0.0)	1 (1.1)	0 (0.0)

^*^ In the 100 mg group. One patient withdrew from the trial before receiving any treatment and was excluded

^†^ One patient has a history of idiopathic hepatitis but had normal liver function test results. Her liver enzyme titers increased since day 7, peaked on day 14, and returned to within the normal range on day 28. She was hospitalized for supportive care for 3 days between day 19 and day 21

^‡^ All seven patients had a history of vigorous exercise before detecting elevated creatine phosphokinase levels. Information about the timing and peaking of creatine phosphokinase values of these seven patients are detailed in Figure S1

^§^ The five patients developed upper respiratory tract symptoms during the follow-up period and recovered smoothly without requiring hospitalization or any antimicrobial agent. All five patients tested negative for COVID-19 using at-home antigen rapid tests

^¶^ One participant detected a breast lump by herself and had a scheduled biopsy before enrollment in this clinical trial

### Exploratory virological endpoints

**Fig. 3:**
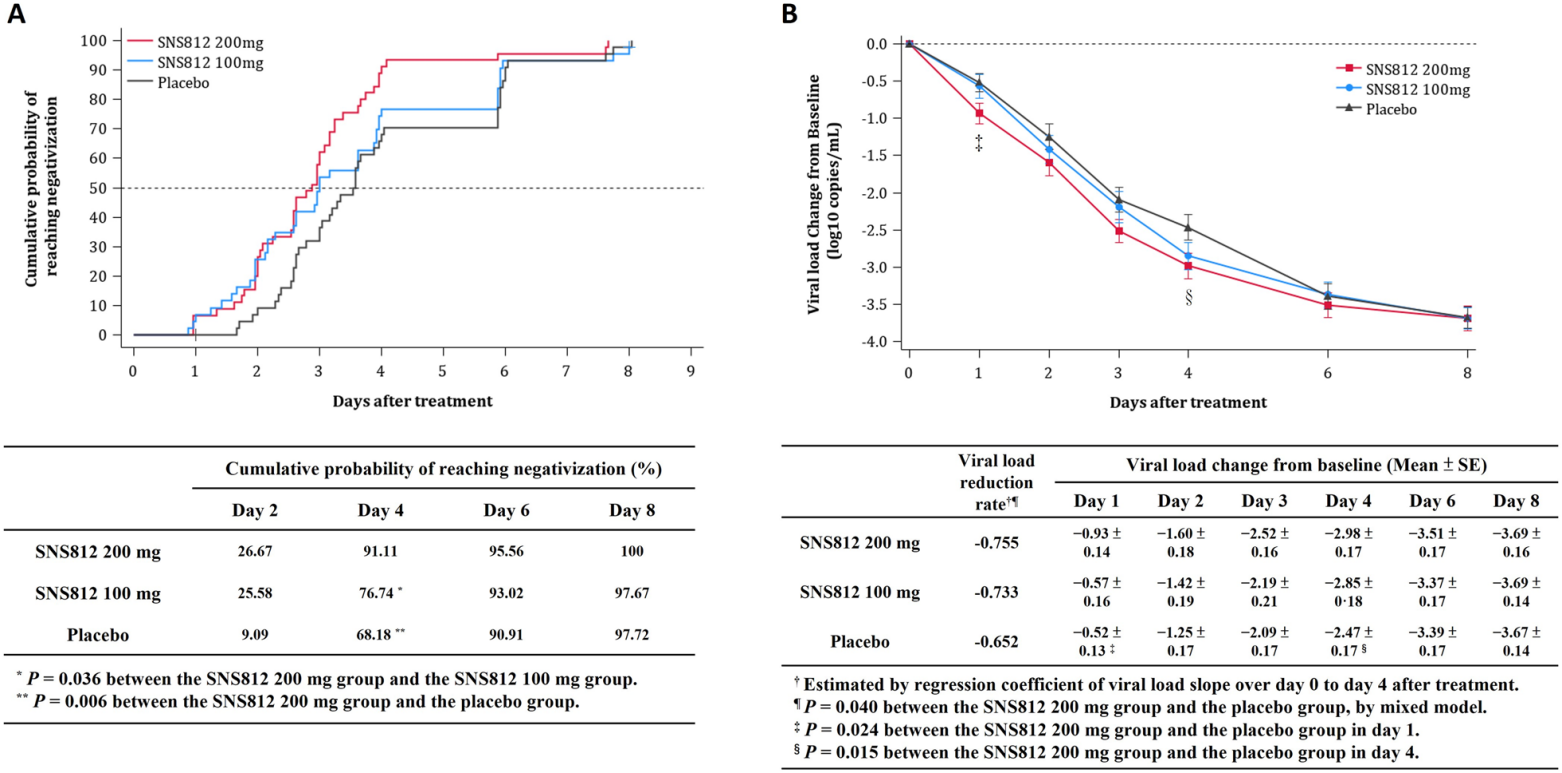
Time to reach negativization of the SARS-CoV-2 antigen test and viral load assessment among 133 patients in the mITT population. **A** shows the Kaplan–Meier Curve of the cumulative probability of achieving SARS-CoV-2 antigen test negativity over time after treatment. On-site SARS-CoV-2 antigen tests with nasopharyngeal swab specimens were performed by research nurses when patients returned to the hospital for treatment or follow-up. All test results were photographed for documentation. Virus negativization was defined as the first day of two consecutive negative test results. **B** shows the viral load changes from baseline over time after treatment. I Bars indicate standard errors. The mean viral loads at baseline on day 1 before dosing were 8.49 ± 1.09 log_10_ copies per milliliter in the SNS812 200 mg group, 8.42 ± 0.87 log_10_ copies per milliliter in the SNS812 100 mg group, and 8.53 ± 0.90 log_10_ copies per milliliter in the placebo group

The median time to SARS-CoV-2 antigen negativity was significantly shorter in the 200-mg SNS812 group than in the placebo group [2.9 days (95% CI 2.54–3.08) vs. 3.6 days (95% CI 3.0–3.96); *p* = 0.007]. The largest between-group difference in antigen negativity was observed on day 4 after treatment, with a higher proportion of participants achieving antigen negativity in the 200-mg SNS812 group than in the 100-mg SNS812 and placebo groups (*p* = 0.036 and *p* = 0.006, respectively) (Fig. 3A). Baseline viral loads were comparable across treatment groups. Changes in viral load from baseline demonstrated a clear dose-dependent response to SNS812. The viral clearance rate, assessed by the regression coefficient of viral load reduction from day 0 to day 4, was significantly greater in the 200-mg SNS812 group than in the placebo group (− 0.755 vs. − 0.652; *p* = 0.040). The reduction in viral load was already significantly greater in the 200-mg SNS812 group by day 1 after treatment initiation and increased further by day 4, resulting in a between-group difference of 0.51 log₁₀ compared with placebo (Fig. 3B).

### Pharmacokinetics, antidrug resistance, and cytokine investigation

Pharmacokinetic analyses demonstrated low systemic exposure to SNS812, with dose-dependent increases in plasma concentrations observed in the 100-mg and 200-mg groups. By day 7 pre-dose, geometric mean concentrations increased to 1.0 ng/mL and 3.5 ng/mL in the 100-mg and 200-mg groups, respectively, and increased further following dosing, consistent with dose-dependent pharmacokinetics (Table S10). Immunogenicity assessments identified one participant in the 100-mg SNS812 group with pre-existing antidrug antibodies detected at all assessed time points, while no treatment-emergent antidrug antibodies were observed in other participants. Cytokine analyses showed no statistically significant differences in CXCL5, G-CSF, or IL-6 levels between placebo and SNS812-treated groups after dosing through day 7, indicating no evidence of SNS812-associated inflammatory cytokine induction.

### Symptom-based endpoints

**Fig. 4:**
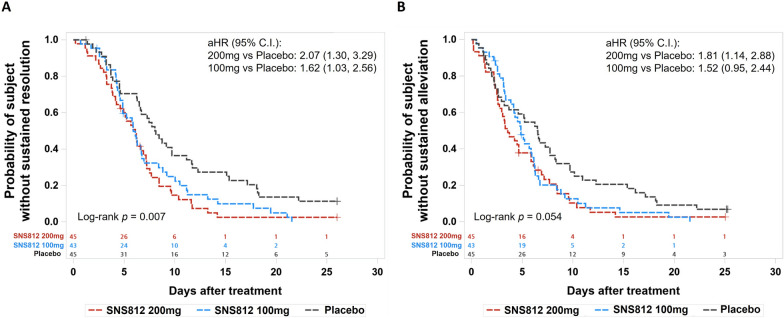
Kaplan–Meier analysis of TTSR and TTSA of all targeted symptoms (mITT population). This analysis evaluates the efficacy of SNS812 in the mITT population by integrating data from nine targeted COVID-19 symptoms, which are as follows: fever, a sore or dry throat, chills or shivers, headache, shortness of breath, nausea, vomiting, loss of smell, and loss of taste. TTSR is shown in the left panel (**A**), and TTSA in the right panel (**B**). *P* value denotes the result of log-rank test. Adjusted hazard ratio (aHR) for treatment groups were adjusted for sex, age (≥ 55 years vs. < 55 years), and the presence of any severe COVID-19 risk factor

No participants progressed to severe COVID-19, required hospitalisation, or died during the study. In the mITT population, the median TTSR for nine prespecified targeted symptoms was significantly shorter in the 200-mg SNS812 group (6.1 days, 95% CI 4.25–7.17) and the 100-mg SNS812 group (5.9 days, 95% CI 4.88–6.75) compared with placebo (8.1 days, 95% CI 6.29–11.17; log-rank *p* = 0.007). After adjustment for sex, age (≥ 55 vs < 55 years), and the presence of any risk factor for severe COVID-19, both the 200-mg (aHR 2.07, 95% CI 1.30–3.29) and 100-mg (aHR 1.62, 95% CI 1.03–2.56) SNS812 groups showed significantly shorter TTSR compared with placebo (Fig. 4A). No significant correlation was observed between viral load reduction and TTSR (Table S11).

In exploratory analyses of TTSR for individual symptoms, 12 of 14 symptoms showed a shorter median time to resolution in the 200-mg SNS812 group compared with placebo. Nominally significant improvements were observed for chills or shivers, loss of taste, shortness of breath, stuffy or runny nose, loss of smell, and nausea in the mITT population and symptomatic subgroups. After Bonferroni correction for multiple comparisons, shortness of breath, loss of taste and chills or shivers remained statistically significant in both the overall mITT population and among participants with the respective symptoms (Figure S2A; Tables S12.1 and S12.2).

When TTSA was assessed, the median TTSA for nine prespecified targeted symptoms was shorter in the 200-mg SNS812 group (3.6 days, 95% CI 2.63–5.88) and the 100-mg SNS812 group (4.9 days, 95% CI 3.88–5.96) than in the placebo group (6.5 days, 95% CI 3.25–8.25; log-rank *p* = 0.054). After covariate adjustment, the 200-mg SNS812 group demonstrated a significantly shorter TTSA compared with placebo (aHR 1.81, 95% CI 1.14–2.88) (Fig. 4B).

Exploratory analyses of TTSA for individual symptoms showed improvements in 11 of 14 symptoms in the 200-mg SNS812 group compared with placebo. Nominal statistical significance was observed for chills or shivers, loss of taste, shortness of breath, and feeling hot or feverish. Following Bonferroni correction, shortness of breath, loss of taste and chills or shivers remained statistically significant in both the overall mITT population and symptomatic subgroups (Figure S2B; Tables S13.1 and S13.2).

## Discussion

SNS812, an aerosol-delivered siRNA antiviral, is a fully chemically modified, carrier-free inhaled siRNA therapeutic and was associated with a favourable safety profile and measurable antiviral activity when initiated within 72 h of symptom onset in vaccinated or previously infected individuals. Treatment was associated with dose-dependent reductions in viral load and shorter time to antigen negativity, with exploratory evidence of accelerated symptom resolution. These results support further clinical investigation of SNS812 as a potential inhaled antiviral candidate for COVID-19, particularly to confirm whether the observed antiviral activity translates into clinically meaningful benefits in larger and higher-risk populations.

Currently available antiviral agents exhibit important limitations, including inconsistent effects on symptom resolution, modest or non-significant reductions in hospitalisation risk—particularly among vaccinated individuals—and constraints imposed by safety concerns and drug–drug interactions [7, 16–19]. Furthermore, recent analyses have identified emergent M_pro_ clinical variants that confer substantial resistance to nirmatrelvir while maintaining protease function, raising concern that treatment-associated selective pressure may further undermine the effectiveness of existing therapeutics [20]. Collectively, these observations highlight the need for next-generation antiviral strategies that provide broad-spectrum activity, maintain efficacy against highly mutating viral populations, and offer favourable tolerability profiles suitable for real-world clinical use.

Safety was the primary objective of this phase 2 study, and the overall safety profile of SNS812 was reassuring. No treatment-related AEs, serious treatment-related AEs, deaths, or AE-related treatment discontinuations were reported. Most TEAEs were mild and largely reflected underlying COVID-19 or comorbidities. Laboratory abnormalities were reviewed individually for temporal association, baseline abnormalities, alternative explanations, and clinical course. The observed liver enzyme elevations occurred in a participant with pre-existing idiopathic hepatitis and resolved spontaneously, while creatine phosphokinase elevations were temporally associated with vigorous exercise during recovery. These abnormalities were isolated, had plausible alternative explanations, and did not suggest a treatment-related safety pattern in hepatic, renal, hematologic, inflammatory, or immunogenicity assessments. Larger studies with broader populations are needed to further characterise the safety profile of SNS812. The safety of SNS812 is likely attributable to the incorporation of 2'-O-methyl, 2'-fluoro, and phosphorothioate modifications, which substantially reduce immune stimulation—both Toll-like receptor-dependent and Toll-like receptor-independent responses—while enhancing molecular stability [12, 13]. Moreover, despite limited pharmacokinetic sampling in this Phase 2 study, SNS812 exhibited dose-dependent exposure consistent with the pharmacokinetic profile characterised in the previous Phase 1 healthy volunteer study [13], supporting the appropriateness of the selected dosing regimen.

The virological findings provide important evidence of rapid biological antiviral activity of SNS812. The dose-dependent reduction in SARS-CoV-2 viral load, observed as early as 24 h after treatment initiation and continuing through day 4, is consistent with the proposed RNAi-mediated mechanism of action and supports target engagement in the respiratory tract. Time to SARS-CoV-2 antigen negativity was also shorter in the 200-mg SNS812 group than in the placebo group, with more than 90% of participants achieving antigen negativity by the fourth dose.

Although viral load reduction and time to antigen negativity are not yet validated surrogates for clinical benefit, they remain clinically relevant supportive endpoints in early-phase antiviral trials. The early timing of viral load reduction observed with SNS812 is notable in the context of previous studies of oral antivirals, including nirmatrelvir and ensitrelvir [17, 19]. However, such cross-trial comparisons are inherently limited by differences in study population, vaccination status, baseline viral load, sampling methods, endpoints, and study design. These findings should therefore be interpreted as supportive evidence of rapid biological antiviral activity rather than comparative clinical efficacy.

The aerosolised route of administration may offer a biologically plausible advantage by delivering siRNA directly to the respiratory tract, the primary site of SARS-CoV-2 replication. Whether this direct respiratory delivery translates into enhanced mucosal antiviral activity or reduced transmission risk warrants further investigation.

Exploratory analyses also indicated potential clinical benefits. Improvements were observed across multiple COVID-19 symptoms, with particularly notable effects in shortness of breath (Figure S3), chills or shivers (Figure S4), loss of smell and loss of taste (Figure S5). Shortness of breath is a well-established predictor of progression to severe disease and intensive-care admission [21, 22]. Its improvement may be clinically relevant, although whether this translates into reduced clinical deterioration requires confirmation in larger, adequately powered trials. Chills or shivers are commonly associated with acute virus-driven systemic inflammatory responses, and their improvement is consistent with attenuation of early innate immune activation following rapid suppression of viral replication. Moreover, the accelerated recovery of loss of smell and taste, which has not been well described in studies of other antivirals, is clinically interesting and may warrant further study, given the relevance of these symptoms to SARS-CoV-2 infection of the upper respiratory and olfactory pathways [23, 24]. However, because TTSA and TTSR were based on patient-reported symptom diaries, these endpoints may be susceptible to inter-individual variability and reporting bias, even in this randomised, controlled trial. Although they are relevant in early-phase outpatient COVID-19 studies, they are less definitive than objective clinical outcomes such as hospitalisation, disease progression, or death. Therefore, the observed symptom-based improvements should be considered exploratory and require confirmation in larger, adequately powered trials.

In this study, we observed that inter-individual variability resulted in instances where reductions in viral load did not consistently correspond with improvements in clinical symptoms. This finding is consistent with previous reports documenting heterogeneous relationships between virological and clinical outcomes [26]. Given this variability, regulatory authorities have noted that virological measures alone may be insufficient to capture clinically meaningful benefit, highlighting the importance of symptom-based and functional endpoints in future trials [14].

This study has several limitations. First, the study population consisted predominantly of younger, vaccinated, non-hospitalised adults with mild to moderate COVID-19 infection. Although 43.0% of participants had at least one risk factor for severe COVID-19, few were older adults and none had moderate or severe disease at baseline. Therefore, the generalisability of these findings to older individuals, patients with substantial comorbidities, and those with moderate or severe COVID-19 remains uncertain and requires evaluation in adequately powered future trials. Second, this phase 2 study had a modest sample size and was powered based on virological change rather than definitive clinical outcomes. Consequently, the study was not designed to evaluate effects on hospitalisation, disease progression, mortality, or other hard clinical endpoints. The symptom-based findings should therefore be considered hypothesis-generating and require confirmation in larger trials. Third, given the prolonged intracellular activity reported for small interfering RNA therapeutics, daily dosing may not be required, and the optimal dosing frequency remains to be determined [25]. Accordingly, future studies are planned to evaluate SNS812 in older and higher-risk populations and to explore alternative dosing regimens informed by pharmacokinetic and virological data.

In conclusion, this phase 2 study showed that inhaled SNS812 was well tolerated in adults with mild-to-moderate COVID-19 and was associated with dose-dependent reductions in viral load, supporting its biological antiviral activity. Exploratory symptom-based analyses suggested potential symptomatic improvement, particularly in the 200-mg group, but these findings should be considered hypothesis-generating. Larger, adequately powered trials in older and higher-risk populations are needed to confirm whether the observed antiviral activity translates into clinically meaningful benefit. This study supports further investigation of inhaled siRNA as a potential antiviral approach for COVID-19.

## Supplementary Information

Supplementary Material 1

## Data Availability

The data that support the findings of this study are not publicly available due to privacy and ethical restrictions. De-identified data may be made available upon reasonable request to the corresponding author, subject to approval by the study sponsor and completion of a data use agreement.
