# Supplementary material for: Safety and antiviral activity of inhaled siRNA SNS812 for treatment of mild-to-moderate COVID-19: a phase 2 randomized trial
Source: J Biomed Sci. 2026 Aug 14;33:83. doi: 10.1186/s12929-026-01284-5 (PMC13474692; doi:10.1186/s12929-026-01284-5)
Supplement: Supplementary file 1 [file 12929_2026_1284_MOESM1_ESM.docx]

**Supplementary Appendix**

**Safety and Efficacy of Inhalable siRNA SNS812 for Mild-to-Moderate COVID-19: A Phase 2 Randomized Trial**

Shey-Ying Chen, M.D., Ph.D. ^1*^, Sui-Yuan Chang, Ph.D.^2*^, Yi-Chung Chang^,^ Ph.D.^3*^, Ming-Che Liu, M.D., Ph.D.^4,5,6,7^, Kuan-Yuan Chen, M.D.^8^, Jer-Hwa Chang, M.D. MSc^9,10^, Wen-Pin Tseng, M.D.^1^, Chien-Hao Lin, M.D.^1^, Jhong-Lin Wu, M.D.^1^, Pai-Chien Chou, M.D., Ph.D.^11,12^, Tsong-Yih Ou, M.D., MSc^13^, Chin-Wang Hsu, M.D.^14,15^, Feng-Yi Chou, B.S.^6^, Hui-Ju Ho, M.S.^3^, Jen Fu Yang M.S.^3^, Chi-Fan Yang, Ph.D.^16^, Yi-Fen Chen Ph.D.^3^, Kang-Yun Lee, M.D., Ph.D.^17†^,William Lu, M.S.^3†^, and Pan-Chyr Yang, M.D., Ph.D.^18‡^

**Affiliations:**

1. Department of Emergency Medicine, National Taiwan University Hospital, College of Medicine, National Taiwan University, Taipei, Taiwan
2. Department of Clinical Laboratory Sciences and Medical Biotechnology, National Taiwan University College of Medicine, Taipei, Taiwan
3. Oneness Biotech Company, Limited, Taipei, Taiwan
4. Department of Urology, Taipei Medical University Hospital, Taipei, Taiwan
5. TMU Research Center of Urology and Kidney, Taipei Medical University, Taipei, Taiwan
6. Clinical Research Center, Taipei Medical University Hospital, Taipei, Taiwan
7. School of Dental Technology, College of Oral Medicine, Taipei Medical University, Taipei, Taiwan
8. Division of Pulmonary Medicine, Department of Internal Medicine, Shuang Ho Hospital, Taipei Medical University, New Taipei City, Taiwan
9. School of Respiratory Therapy, College of Medicine, Taipei Medical University, Taipei, Taiwan
10. Division of Pulmonary Medicine, Department of Internal Medicine, Wan Fang Hospital, Taipei Medical University, Taipei, Taiwan
11. Division of Pulmonary Medicine, Department of Internal Medicine, Taipei Medical University Hospital, Taipei, Taiwan
12. Division of Thoracic Medicine, Department of Internal Medicine, School of Medicine, College of Medicine, Taipei Medical University, Taipei, Taiwan
13. Division of Infectious Diseases, Department of Internal Medicine, Wan Fang Hospital, Taipei Medical University, Taipei, Taiwan
14. Department of Emergency, School of Medicine, College of Medicine, Taipei Medical University, Taipei, Taiwan
15. Emergency Department, Department of Emergency and Critical Medicine, Wan Fang Hospital, Taipei Medical University, Taipei, Taiwan
16. Microbio (Shanghai) Biotech Company, Shanghai, China
17. Division of Pulmonary Medicine, Department of Internal Medicine, School of Medicine, College of Medicine, Taipei Medical University, Taipei, Taiwan
18. Department of Internal Medicine, National Taiwan University College of Medicine, Taipei, Taiwan, pcyang@ntu.edu.tw

**Table of Contents**

[Appendix 1. List of Investigators and Data and Safety Monitoring Board (DSMB)	5](#_Toc218974159)

[Appendix 2. Supplementary Methods	7](#_Toc218974160)

[Study Responsibilities	7](#_Toc218974161)

[Study Design	7](#_Toc218974162)

[Case Number Estimation	7](#_Toc218974163)

[Dose Selection	7](#_Toc218974165)

[Aerosol Characterisation and Nebuliser Compatibility	8](#_Toc218974167)

[Eligibility Criteria	8](#_Toc218974168)

[Viral Sampling, Lineage, and Load Assessment	8](#_Toc218974170)

[Appendix 3. Other Supplementary Results	10](#_Toc218974174)

[Table S1. COVID-19 severity categorization and definition	10](#_Toc218974175)

[Table S2. Aerosol delivery devices and aerosol characterisation across the development stages of SNS812.	12](#_Toc218974176)

[Table S3. Symptoms attributable to COVID-19 collected and analyzed for efficacy evaluation	13](#_Toc218974179)

[Table S4. The 14 COVID-19-related symptoms and associated 4-point severity assessment scale	14](#_Toc218974180)

[Table S5. Statistical analysis populations	15](#_Toc218974181)

[Table S6. Factors associated with the development of severe COVID-19	16](#_Toc218974182)

[Table S7. Complete viral genotype identified from the 135 patients	18](#_Toc218974183)

[Table S8. Demographic and clinical characteristics of the participants with treatment-emergent adverse event	20](#_Toc218974184)

[Table S9. Incidence of treatment emergent adverse events (TEAEs), by age (SS)	......................................................................................................................................22](#_Toc218974185)

[Table S10. Summary of pharmacokinetics (PKS) investigation	23](#_Toc218974186)

[Table S11. Spearman correlation results between viral load reduction and time to sustained resolution of targeted COVID-19 symptoms	24](#_Toc218974187)

[Table S12. Time to sustained resolution of individual COVID-19 symptoms, by modified intention-to-treat Set (Table S7.1), and by patients with symptom in each group (Table S7.2)	25](#_Toc218974188)

[Table S13. Time to sustained alleviation of individual COVID-19 symptoms, by modified intention-to-treat Set (Table S8.1), and by patients with symptom in each group (Table S8.2)	33](#_Toc218974189)

[Figure S1. Timing and peak value of creatine kinase (CK) elevation in seven participants	41](#_Toc218974190)

[Figure S2. Improvement of individual COVID-19 symptoms following treatment with 200 mg SNS812 (mITT: participants with the symptom)	41](#_Toc218974190)

[Figure S3. Kaplan–Meier analysis of time to sustained resolution and alleviation of shortness of breath (mITT population)	44](#_Toc218974191)

[Figure S4. Kaplan–Meier analysis of time to sustained resolution and alleviation of chills and shivers (mITT population)	45](#_Toc218974192)

[Figure S5. Kaplan–Meier analysis of time to sustained resolution and alleviation of loss of taste and smell (mITT population)	46](#_Toc218974193)

[Appendix 4 Summary of Safety Evaluation from CSR	47](#_Toc218974195)

[Overall Summary of Safety Evaluation	47](#_Toc218974196)

[1.1.	AEs	47](#_Toc218974197)

[**1.1.1.**	**Brief Summary of the AEs**	47](#_Toc218974198)

[**1.1.2.**	Display of the TEAEs	49](#_Toc218974199)

[**1.1.3.**	Brief Summary of the TRAEs	52](#_Toc218974200)

[1.2.	Deaths, Other SAEs, and Other Significant AEs	52](#_Toc218974201)

[**1.2.1.**	Listing of Deaths, Other SAEs, and Other Significant AEs	52](#_Toc218974202)

[**1.2.2.**	Narrative of Other SAEs	52](#_Toc218974203)

[1.3.	Clinical Laboratory Evaluation	53](#_Toc218974204)

[**1.3.1.**	Hematology	53](#_Toc218974205)

[**1.3.2.**	Biochemistry	56](#_Toc218974206)

[**1.3.3.**	Coagulation Indices	62](#_Toc218974207)

[**1.3.4.**	Urinalysis	62](#_Toc218974208)

[1.4.	Vital Signs, Physical Findings, and Other Observations Related to Safety	63](#_Toc218974209)

[**1.4.1.**	Vital Signs	63](#_Toc218974210)

[**1.4.2.**	12-Lead ECG	63](#_Toc218974211)

[**1.4.3.**	Chest X-Ray	63](#_Toc218974212)

[**1.4.4.**	SpO_2_ and the Respiratory Support Status	63](#_Toc218974213)

[1.5.	Safety Conclusions	63](#_Toc218974214)

# Appendix 1. List of Investigators and Data and Safety Monitoring Board (DSMB)

Study sites and investigators

| **Name** | **Institute** | **Location** |
| --- | --- | --- |
| Shey-Ying Chen  Wen-Pin Tseng  Jhong-Lin Wu  Chien-Hao Lin  Wang-Huei Sheng  Chyi-Feng Jan  Chen-Chi Wu  Shao-Yi Cheng  Wen-Chien Chou  Chia-Lin Tseng  Tsu-Yao Cheng  Hao-Hsaing Chang | National Taiwan University Hospital | Taipei, Taiwan |
| Chi-Ying Lin | National Taiwan University Hospital Yunlin Branch | Yunlin County, Taiwan |
| Han-Yueh Kuo | National Taiwan University Hsin-Chu Hospital | HsinChu City, Taiwan |
| Barbara Rizzardi | Velocity Clinical Research | Utah, USA |
| Kuan-Yuan Chen  Kang-Yun Lee  Po-Hao Feng  Jing-Quan Zheng | Taipei Medical University – Shuang Ho Hospital, Ministry of Health and Welfare | New Taipei City, Taiwan |
| Ming-Che Liu  Pai-Chien Chou  Chi-Li Chung  Kai-Ling Lee  Mei-Chuan Chen  Sen-Kuang Hou | Taipei Medical University Hospital | Taipei, Taiwan |
| Jer-Hwa Chang  Chin-Wang Hsu  Tsong-Yih Ou  Fu-Lun Chen  Ying-Chin Lin  Yang-Ching Chen  Chun-Jui Chang  Yi-Hsuan Tsai  Cheng-Ching Lin  Chih-Yin Wu  Sheng-Lun Hsu | Taipei Municipal Wanfang Hospital | Taipei, Taiwan |

Members of the Independent Data and Safety Monitoring Committee

| Name | Role | Affiliations | Title | Email |
| --- | --- | --- | --- | --- |
| Chun-Hsing Liao | Chairperson | Far Eastern Memorial Hospital | MD. | liaochunhsing@gmail.com |
| Yi-Tzu Lee | Clinician | Taipei Veterans General Hospital | MD. | yitzulee@gmail.com |
| Chin-Hao Chang | Statistician | National Taiwan University Hospital | Dr. | chinhao@ntuh.gov.tw |

# Appendix 2. Supplementary Methods

## Study Responsibilities

The principal investigators and Oneness Biotech were responsible for the study design. Oneness Biotech was responsible for study conduct, data collection, analysis, and interpretation. The principal investigators and Oneness Biotech jointly took responsibility for writing this manuscript. SNS812 was manufactured by WuXi STA Pharmaceutical Co., Ltd. The placebo, normal saline, was manufactured by Taiwan Biotech Co., Ltd. All data were available to all authors, who vouch for the accuracy and completeness of this report, as well as the study’s adherence to the protocol.

## Study Design

## Case Number Estimation

## The sample size calculation was based on the endpoint of change from baseline in viral load, considering three treatment arms. It was assumed that the mean reduction was –2.4 for the treatment group compared to –1.5 for the placebo group, with a common standard deviation of 1.3. Using a Wilcoxon rank-sum test with a two-sided type I error of 0.05 and a power of 0.80, the required sample size was estimated to be 38 participants per group. After accounting for an anticipated 15% dropout rate, the planned enrollment is approximately 45 participants per group, resulting in a total of 135 participants across the three study arms.

## Dose Selection

## According to the completed clinical trial (SNS812CLCT01), MBS-COV was safe and well tolerated in healthy participants within the dose range of 0.3 mg/kg to 1.2 mg/kg. Due to the complexity of calculating individual patient doses based on body weight (mg/kg) and the associated administration process, a fixed-dose administration method was designed for Phase 2 to improve treatment compliance and better align with post-marketing use. Two fixed doses within the maximum tolerated dose (MTD) range were selected: 100 mg (approximately 0.5 mg/kg) and 200 mg (approximately 1.0 mg/kg). These doses were used to evaluate the safety, tolerability, efficacy, and pharmacokinetics of SNS812 in participants with mild-to-moderate COVID-19.

## Aerosol Characterisation and Nebuliser Compatibility

Aerosol performance of SNS812 was assessed prior to clinical use by an independent contract research organisation using standard cascade impaction methods. Aerosol parameters, including mass median aerodynamic diameter, geometric standard deviation, and fine particle fraction, were verified to meet predefined criteria for respirable lower-airway delivery (Table S2). In addition, the mesh nebuliser used in this study was evaluated for device compatibility through more than 30 consecutive dosing cycles, with no evidence of device clogging or deterioration in aerosol output.

## Eligibility Criteria

## Eligible participants were aged ≥18 years, had mild-to-moderate SARS-CoV-2 infection confirmed by reverse transcription polymerase chain reaction (RT-PCR), body mass index between 18.0 and 32.0 kg/m^2^, and demonstrated at least one clinical manifestation of COVID-19 within 3 days before receiving SNS812 or placebo. Key exclusion criteria were anticipated progression to severe COVID-19, hospitalization requirement, active smoker, severe cardiovascular, neurological, lung, hepatic, or renal disease, receiving systemic steroid, previous SARS-CoV-2 monoclonal antibody or antiviral treatment within 30 days, or receipt of any vaccine, COVID-19 immunoglobulin, or convalescent plasma within 3 months. Full eligibility criteria are available at ClinicalTrials.gov (NCT05941793)

# Viral Sampling, Lineage, and Load Assessment

# All samples for SARS-CoV-2 antigen and nucleic acid testing were collected using COPAN UTM (330C) and nasopharyngeal swabs (503CS01), respectively, by study nurses following standard collection protocols at each trial site. Samples for viral load determination were collected during the screening phase; on days 1, 5, 7, 9, 14, and 28 (once daily); and on days 2, 3, and 4 (twice daily), with a sampling interval of more than 12 ± 3 h. Samples for assessing viral antigen negativity were obtained alongside nucleic acid tests until two consecutive negative results were confirmed.

# Nasopharyngeal swab specimens were aliquoted for viral RNA extraction using the QIAamp Viral RNA Kit (52904, QIAGEN, Germany). The extracted RNA was then used for library construction for Illumina sequencing with the Illumina COVIDSeq Assay Kit (Illumina, CA). After sequencing, raw data underwent quality control to remove low-quality reads and adapter sequences. The cleaned reads were aligned to the SARS-CoV-2 reference genome. To determine the viral lineage, the UShER tool (Ultrafast Sample Placement on Existing tRees) was used to place sequences into a global phylogenetic reference tree of known SARS-CoV-2 lineages. This enabled accurate classification of each sequence into its specific sublineage, including Omicron variants.

# Quantitative viral load was measured using a validated real-time qPCR assay conducted at a central laboratory. The assay, developed by TFBS Bioscience (Taipei, Taiwan), targeted the envelope (E) gene and was performed using the Applied Biosystems 7500 Fast System. Assay validation followed U.S. FDA guidelines. For specimens with viral loads below the lower limit of quantification (LLOQ), defined as log₁₀ (1.00 × 10⁵ copies/mL), values were imputed as log₁₀ (LLOQ/2 copies/mL) to provide a conservative estimate. A mixed-effects model was used to compare changes in viral load from baseline to day 28 between study groups.

# Appendix 3. Other Supplementary Results

## Table S1. COVID-19 severity categorization and definition

| **Category** | **Definition** |
| --- | --- |
| SARS-CoV-2 infection without symptoms | - Positive result from a virologic test (i.e., a nucleic acid amplification test or an antigen test) - No symptoms |
| Mild COVID-19 | - Positive result from a virologic test (i.e., a nucleic acid amplification test or an antigen test) - Symptoms of mild illness, which may include fever, cough, sore throat, malaise, headache, muscle pain, nausea, vomiting, diarrhea, or loss of taste or smell, without shortness of breath or dyspnea - No clinical signs indicative of moderate, severe, or critical disease |
| Moderate COVID-19 | - Positive result from a virologic test (i.e., a nucleic acid amplification test or an antigen test) - Symptoms of moderate illness, which may include any symptom of mild illness or shortness of breath with exertion - Clinical signs suggestive of moderate illness, such as respiratory rate ≥ 20 breaths per minute, heart rate ≥ 90 beats per minute, and oxygen saturation (SpO₂) > 93% on room air at sea level - No clinical signs indicative of severe or critical disease |
| Severe COVID-19 | - Positive result from a virologic test (i.e., a nucleic acid amplification test or an antigen test) - Symptoms suggestive of severe systemic illness, which may include any symptom of moderate illness, shortness of breath at rest, or respiratory distress - Clinical signs indicative of severe systemic illness, such as respiratory rate ≥ 30 breaths per minute, heart rate ≥ 125 beats per minute, SpO_2_ ≤ 93% on room air at sea level, or PaO_2_/FiO_2_ < 300 - No clinical criteria for critical disease |
| Critical COVID-19 | - Positive result from a virologic test (i.e., a nucleic acid amplification test or an antigen test) - Evidence of critical illness, defined by at least one of the following:   - Respiratory failure requiring at least one of the following:     - Endotracheal intubation and mechanical ventilation, oxygen delivered by high-flow nasal cannula (heated, humidified oxygen via reinforced nasal cannula at flow rates > 20 L/min with a fraction of inspired oxygen ≥ 0.5), noninvasive positive pressure ventilation, extracorporeal membrane oxygenation   - Shock (defined by systolic blood pressure < 90 mm Hg, diastolic blood pressure < 60 mm Hg, or requiring vasopressors)   - Multi-organ dysfunction or failure |

## Table S2. Aerosol delivery devices and aerosol characterisation across the development stages of SNS812.

| **Stage** | **Nebuliser** | **CRO** | **MMAD (µm)** | **GSD** | **FPF**  **(<5 µm, %)** |
| --- | --- | --- | --- | --- | --- |
| **Discovery** | Aeroneb® (Aerogen Ltd., Galway, Ireland) | Independent CRO | 4.725 | 2.376 | 51.94 |
| **Preclinical** | Air Pro III® (Feellife Health Inc., China) | Independent CRO | 4.498 | 1.801 | 55.75 |
| **Phase 1** | Air Pro III® (Feellife Health Inc., China) | Independent, GLP-compliant CRO | 4.553 | 1.684 | 56.35 |
| **Phase 2** |  |  | 4.458 | 1.684 | 58.25 |

## Note: All Aerosol characterisation was performed using standard cascade impaction methods by independent CRO specialising in inhalation testing. Values are presented as measured under the specified testing conditions for each device and development stage.

## MMAD: mass median aerodynamic diameter; GSD: geometric standard deviation; FPF: fine particle fraction (percentage of particles <5 µm); CRO: contract research organisation; GLP: Good Laboratory Practice.

## Table S3. Symptoms attributable to COVID-19 collected and analyzed for efficacy evaluation

| **Symptom** | **Daily symptoms collected (by study protocol)** | **Targeted symptoms (by study protocol)** | **Symptoms showing trend of dose-dependent improvement (analysis results after deblending)** |
| --- | --- | --- | --- |
| 1. Feeling hot or feverish | X | X | X |
| 2. Cough | X |  |  |
| 3. Muscle or body ache | X |  |  |
| 4. Sore or dry throat | X | X | X |
| 5. Low energy or tiredness | X |  | X |
| 6. Chills or shivers | X | X | X |
| 7. Stuffy or runny nose | X |  | X |
| 8. Headache | X | X | X |
| 9. Shortness of breath (difficulty breathing) | X | X | X |
| 10. Nausea (feeling like you might throw up) | X | X |  |
| 11. Vomiting | X | X |  |
| 12. Diarrhea | X |  |  |
| 13. Loss of smell | X | X | X |
| 14. Loss of taste | X | X | X |

## Table S4. The 14 COVID-19-related symptoms and associated 4-point severity assessment scale

| **Symptoms** | **Score** |
| --- | --- |
| 1. Feeling hot or feverish | None = 0 points  Mild = 1 point  Moderate = 2 points  Severe = 3 points  Note: For symptoms 1–10, the question may be phrased: “How serious has it been in the last 12 or 24 h?” |
| 2. Cough |  |
| 3. Muscle or body aches |  |
| 4. Sore or dry throat |  |
| 5. Low energy or tiredness |  |
| 6. Chills or shivers |  |
| 7. Stuffy or runny nose |  |
| 8. Headache |  |
| 9. Shortness of breath (difficulty breathing) |  |
| 10. Nausea (feeling like you need to vomit) |  |
| 11. Vomiting | None = 0 points  1–2 times = 1 point  3–4 times = 2 points  More than 5 times = 3 points  Note: The question may be phrased: “How many times have you vomited in the last 12 or 24 h?” |
| 12. Diarrhea | None = 0 points  1–2 times = 1 point  3–4 times = 2 points  More than 5 times = 3 points  Note: The question may be phrased: “How many times have you had diarrhea in the last 12 or 24 h?” |
| 13. Loss of smell | Normal sense of smell = 0 points  Reduced sense of smell = 1 point  Complete loss of smell = 2 points  Note: The question may be phrased: “How has your sense of taste been in the last 12 or 24 h?” |
| 14. Loss of taste | Normal sense of taste = 0 points  Reduced sense of taste = 1 point  Complete loss of taste = 2 points  Note: The question may be phrased: “How has your sense of taste been in the last 12 or 24 h?” |

## Table S5. Statistical analysis populations

| **Population** | **Description** |
| --- | --- |
| **Full analysis set (FAS)** | All randomized participants. |
| **Modified Intention-to-Treat (mITT)** | All randomized participants without any medical history that may impact patient-reported outcomes, who received at least one study drug, performed baseline assessments, and had at least one post-baseline SARS-CoV-2 antigen test evaluation. |
| **Per Protocol Set (PPS)** | A subset of the FAS, including participants with evaluable data and no significant protocol deviations. |
| **Safety Set (SS)** | All randomized participants who received at least one study drug. |
| **Pharmacokinetics Set (PKS)** | All randomized participants who received at least one dose of the study drug and have at least one plasma concentration measurement. |
| **Immunogenicity Set (IS)** | All randomized participants who received at least one dose of the study drug and have at least one anti-drug antibody (ADA) concentration measurement. |

**Note:** The FAS analysis set was used for the disposition and demographic baseline characteristics analysis. Both the mITT and PPS analysis sets were used in the analyses of the efficacy endpoints. The Safety Analysis Set was used in the analyses of the safety data. The PKS was used for pharmacokinetics analysis. The IS was used for immunogenicity analysis.

## Table S6. Factors associated with the development of severe COVID-19

| **Risk Factor** | **Description** |
| --- | --- |
| **Conclusive High Risk** | |
| Age | ≥65 years |
| Medical conditions | Asthma; Cancer (hematological malignancy); Cerebrovascular disease; Chronic kidney disease or receiving dialysis; Chronic lung diseases (limited to bronchiectasis, chronic obstructive pulmonary disease, interstitial lung disease, pulmonary embolism, pulmonary hypertension); Chronic liver diseases (limited to cirrhosis, non-alcoholic fatty liver disease, alcoholic liver disease, autoimmune hepatitis); Cystic fibrosis; Diabetes mellitus (type 1 and type 2); Heart conditions (heart failure, coronary artery disease, cardiomyopathy); Human immunodeficiency virus infection; Dementia; Obesity (BMI ≥ 30 kg/m^2^ or ≥ 95th percentile in children); Physical inactivity; Pregnancy and recent pregnancy; Primary immunodeficiencies; Smoking (current and former); Solid organ or blood stem cell transplantation; Tuberculosis; Use of corticosteroids or other immunosuppressive medications. |
| Disabilities | Attention-deficit/hyperactivity disorder (ADHD); Autism; Charcot foot; Chromosomal disorders; Chromosome 17 and 19 deletion; Chromosome 18q deletion; Cognitive impairment; Congenital hydrocephalus; Congenital malformations; Deafness/hearing loss; Disability indicated by Barthel Index; Down syndrome; Fahr’s syndrome; Fragile X syndrome; Gaucher disease; Hand and foot disorders; Learning disabilities; Leber’s hereditary optic neuropathy (LHON) or autosomal dominant optic atrophy (ADOA); Leigh syndrome; Limitations with self-care or activities of daily living; Maternal inherited diabetes and deafness (MIDD); Mitochondrial encephalopathy, lactic acidosis, and stroke-like episodes (MELAS) and risk markers; Mobility disability; Movement disorders; Multiple disabilities (referred to in research papers as “bedridden disability”); Multisystem disease; Myoclonic epilepsy with ragged red fibers (MERRF); Myotonic dystrophy; Neurodevelopmental disorders; Neuromuscular disorders; Neuromyelitis optica spectrum disorder (NMOSD); Neuropathy, ataxia, and retinitis pigmentosa (NARP); Perinatal spastic hemiparesis; Primary mitochondrial myopathy (PMM); Progressive supranuclear palsy; Senior-Loken syndrome; Severe and complex disability (referred to in research papers as “polyhandicap disability”); Spina bifida and other nervous system anomalies; Spinal cord injury; Tourette syndrome; Traumatic brain injury; Visual impairment/blindness; Wheelchair use. |
| Mental health conditions | Mood disorders (including depression); Schizophrenia spectrum disorders. |
| **Suggestive High Risk** | |
| Medical conditions | Overweight (BMI >25 kg/m^2^ but <30 kg/m^2^); Sickle cell disease; Substance use disorders. |
| Mixed Evidence | |
| Medical conditions | Alpha-1 antitrypsin deficiency; Bronchopulmonary dysplasia; Hepatitis B; Hepatitis C; Hypertension; Thalassemia. |

## Table S7. Complete viral genotype identified from the 135 patients

| **SARS-CoV-2 strain genotype – No. (%)** | **SNS812 200 mg (N = 45)** | | **SNS812 100 mg (N = 45)** | | **Placebo (N = 45)** | | **Total (N = 135)** | |
| --- | --- | --- | --- | --- | --- | --- | --- | --- |
| Omicron (BA.2-like) |  |  |  |  |  |  |  |  |
| JN.1 | 11 | (24.4) | 11 | (24.4) | 10 | (22.2) | 32 | (23.7) |
| JN.1.1 | 1 | (2.2) | 1 | (2.2) | 1 | (2.2) | 3 | (2.2) |
| JN.1.16 | 3 | (6.7) | 1 | (2.2) | 1 | (2.2) | 5 | (3.7) |
| JN.1.20 | 0 | (0.0) | 1 | (2.2) | 1 | (2.2) | 2 | (1.5) |
| JN.1.32 | 0 | (0.0) | 1 | (2.2) | 0 | (0.0) | 1 | (0.7) |
| JN.1.37 | 0 | (0.0) | 1 | (2.2) | 1 | (2.2) | 2 | (1.5) |
| JN.1.39.3 | 1 | (2.2) | 0 | (0.0) | 0 | (0.0) | 1 | (0.7) |
| JN.1.4 | 2 | (4.4) | 3 | (6.7) | 2 | (4.4) | 7 | (5.2) |
| JN.1.4.5 | 1 | (2.2) | 3 | (6.7) | 2 | (4.4) | 6 | (4.4) |
| JN.1.5 | 0 | (0.0) | 3 | (6.7) | 1 | (2.2) | 4 | (3.0) |
| JN.1.57 | 0 | (0.0) | 0 | (0.0) | 1 | (2.2) | 1 | (0.7) |
| JN.1.57.1 | 1 | (2.2) | 0 | (0.0) | 1 | (2.2) | 2 | (1.5) |
| JN.1.7 | 0 | (0.0) | 0 | (0.0) | 1 | (2.2) | 1 | (0.7) |
| KP.1 | 1 | (2.2) | 0 | (0.0) | 2 | (4.4) | 3 | (2.2) |
| KP.1.1 | 2 | (4.4) | 1 | (2.2) | 1 | (2.2) | 4 | (3.0) |
| KP.1.1.3 | 1 | (2.2) | 0 | (0.0) | 0 | (0.0) | 1 | (0.7) |
| KP.2 | 2 | (4.4) | 1 | (2.2) | 3 | (6.7) | 6 | (4.4) |
| KP.2.1 | 0 | (0.0) | 1 | (2.2) | 0 | (0.0) | 1 | (0.7) |
| KP.2.10 | 0 | (0.0) | 0 | (0.0) | 1 | (2.2) | 1 | (0.7) |
| KP.2.2 | 2 | (4.4) | 1 | (2.2) | 0 | (0.0) | 3 | (2.2) |
| KP.2.3 | 1 | (2.2) | 2 | (4.4) | 0 | (0.0) | 3 | (2.2) |
| KP.2.3.2 | 0 | (0.0) | 0 | (0.0) | 1 | (2.2) | 1 | (0.7) |
| KP.3 | 0 | (0.0) | 0 | (0.0) | 1 | (2.2) | 1 | (0.7) |
| KP.3.2 | 0 | (0.0) | 1 | (2.2) | 0 | (0.0) | 1 | (0.7) |
| KP.3.3 | 1 | (2.2) | 2 | (4.4) | 2 | (4.4) | 5 | (3.7) |
| KP.4.1.2 | 1 | (2.22) | 0 | (0.0) | 0 | (0.0) | 1 | (0.7) |
| LB.1 | 1 | (2.22) | 0 | (0.0) | 1 | (2.2) | 2 | (1.5) |
| LB.1.4 | 0 | (0.0) | 1 | (2.2) | 0 | (0.0) | 1 | (0.7) |
| LB.1.5 | 0 | (0.0) | 2 | (4.4) | 2 | (4.4) | 4 | (3.0) |
| LB.1.7 | 8 | (17.8) | 2 | (4.4) | 6 | (13.3) | 16 | (11.9) |
| LJ.1 | 1 | (2.2) | 1 | (2.2) | 1 | (2.2) | 3 | (2.2) |
| XDQ.1 | 0 | (0.0) | 0 | (0.0) | 1 | (2.2) | 1 | (0.7) |
| XDV.1 | 1 | (2.2) | 3 | (6.7) | 0 | (0.0) | 4 | (3.0) |
| Omicron (XBB-like) |  |  |  |  |  |  |  |  |
| HK.3 | 2 | (4.4) | 0 | (0.0) | 1 | (2.2) | 3 | (2.2) |
| Omicron (XBB.1-like) |  |  |  |  |  |  |  |  |
| EG.5.1 | 1 | (2.2) | 1 | (2.2) | 0 | (0.0) | 2 | (1.5) |
| Omicron (XBB.1.5-like) |  |  |  |  |  |  |  |  |
| JD.1.1.3 | 0 | (0.0) | 1 | (2.2) | 0 | (0.0) | 1 | (0.7) |

## Table S8. Demographic and clinical characteristics of the participants with treatment-emergent adverse event

| **Characteristics** | **SNS812 200 mg**  **(N = 27)** | **SNS812 100 mg**  **(N = 24)** | **Placebo**  **(N = 21)** | **Total**  **(N = 72)** |
| --- | --- | --- | --- | --- |
| Median age (range) – yr | 34 (19–67) | 33.5 (23–60) | 36 (25–66) | 34 (19–67) |
| Male sex – no. (%) | 12 (44.4) | 10 (41.7) | 7 (33.3) | 29 (40.3) |
| Current smoker | 0 (0.0) | 0 (0.0) | 0 (0.0) | 0 (0.0) |
| Risk factors for severe Covid-19 – no. (%) |  |  |  |  |
| At least one risk factor | 12 (44.4) | 15 (62.5) | 7 (33.3) | 34 (47.2) |
| Age ≧ 65 yr | 1 (3.7) | 0 (0.0) | 2 (9.5) | 3 (4.2) |
| Overweight or obesity* | 11 (40.7) | 11 (45.8) | 6(28.6) | 28 (38.9) |
| Overweight | 10 (37.0) | 7 (29.2) | 6 (28.6) | 23 (31.9) |
| Obesity | 1 (3.7) | 4 (16.7) | 0 (0.0) | 5 (6.9) |
| Diabetes mellitus | 1 (3.7) | 1 (4.2) | 1 (4.8) | 3 (4.2) |
| Hypertension | 4 (14.8) | 3 (12.5) | 2 (9.5) | 9 (12.5) |
| Coronary artery disease | 0 (0.0) | 0 (0.0) | 1 (4.8) | 1 (1.4) |
| Chronic liver disease† | 0 (0.0) | 1 (4.2) | 0 (0.0) | 1 (1.4) |
| Asthma | 0 (0.0) | 2 (8.3) | 1 (4.8) | 3 (4.2) |
| Depression | 0 (0.0) | 0 (0.0) | 0 (0.0) | 0 (0.0) |
| Chronic kidney disease | 1 (3.7)‡ | 0 (0.0) | 0 (0.0) | 1 (1.4) |
| Thalassemia | 1 (3.7) | 0 (0.0) | 0 (0.0) | 1 (1.4) |
| Covid-19 vaccination within 3 months | 0 (0.0) | 0 (0.0) | 0 (0.0) | 0 (0.0) |
| Covid-19 severity in screening – no. (%) |  |  |  |  |
| Mild | 27 (100.0) | 24 (100.0) | 21 (100.0) | 72 (100.0) |
| Moderate§ | 0 (0.0) | 0 (0.0) | 0 (0.0) | 0 (0.0) |
| Median total score for the 14 symptoms at enrollment (range)¶,¥ | 11 (5–20) | 11 (4–25) | 13 (4–17) | 11.5 (4–25) |

* Overweight was defined as a body-mass index (BMI, the weight in kilograms divided by the square of the height in meters) of 25 kg/m^2^ to 29 kg/m^2^, and obesity was defined as a BMI of ≥30 kg/m^2^ on the day of eligibility evaluation.

† Includes one case of hepatic steatosis.

‡ The patient had a history of idiopathic nephropathy; however, he had normal renal function on enrollment.

¶ Includes fever, cough, muscle or body aches, sore or dry throat, low energy or tiredness, chills or shivers, stuffy or runny nose, headache, shortness of breath or difficulty breathing, nausea, vomiting, diarrhea, loss of smell and loss of taste.

¥ Patients assessed the severity of each of the 14 COVID-19-associated symptoms on a four-point scale from 0 to 3, with zero indicating no symptoms, 1 mild symptoms, 2 moderate symptoms, and 3 severe symptoms.

## Table S9. Incidence of treatment emergent adverse events (TEAEs), by age (SS)

| **Age group**  **Characteristics** | **SNS812 200 mg**  **(N = 45)** | **SNS812 100 mg**  **(N = 44)** | **Placebo group**  **(N = 45)** | **Total**  **(N = 134)** | **P-value** |
| --- | --- | --- | --- | --- | --- |
| **Age ≥ 55 yr**  Number of patients  Number of Patients with TEAEs  Incidence (%) | 7  6  85.71% | 3  2  66.67% | 8  6  75.00% | 18  14  77.78% | >0.999 |
| **Age < 55 yr**  Number of patients  Number of Patients with TEAEs  Incidence (%) | 38  21  55.26% | 41  22  53.66% | 37  15  40.54% | 116  58  50.00% | 0.406 |
| ***P*-value#** | 0.215 | >0.999 | 0.121 | 0.041* |  |

***P*-value was performed by Fisher-exact test.**

**# *P*-values compare AE incidence between ≥55 and <55 years within each treatment group**

***Statistically significant**

## Table S10. Summary of pharmacokinetics (PKS) investigation

| **Visit** | **SNS812 200 mg**  **(N = 45)** | **SNS812 100 mg**  **(N = 45)** | **Placebo**  **(N = 45)** |
| --- | --- | --- | --- |
| Day 1 (pre-dose) |  |  |  |
| N | 45 | 44 | 45 |
| Mean (SD) (ng/mL) | 0.4 (0.65) | 1.3 (5.86) | 0.4 (1.07) |
| Geometric mean (ng/mL) | 0.2 | 0.2 | 0.1 |
| Geometric CV (%) | 310 | 410 | 230 |
| Range | (0.0, 3.2) | (0.0, 39.0) | (0.0, 6.8) |
| Day 7 (pre-dose) |  |  |  |
| N | 44 | 41 | 43 |
| Mean (SD) (ng/mL) | 6.1 (6.66) | 1.6 (1.36) | 0.4 (0.43) |
| Geometric mean (ng/mL) | 3.5 | 1 | 0.2 |
| Geometric CV (%) | 160 | 150 | 180 |
| Range | (0.3, 33.8) | (0.0, 5.7) | (0.0, 2.2) |
| Day 7 (post-dose 1 h) |  |  |  |
| N | 44 | 42 | 44 |
| Mean (SD) (ng/mL) | 35.3 (30.51) | 14.6 (10.28) | 0.6 (1.37) |
| Geometric mean (ng/mL) | 26.2 | 11.3 | 0.2 |
| Geometric CV (%) | 110 | 100 | 360 |
| Range | (0.7, 184.2) | (1.0, 49.0) | (0.0, 8.8) |
| Day 7 (post-dose 2 h) |  |  |  |
| N | 44 | 42 | 44 |
| Mean (SD) (ng/mL) | 41.0 (43.87) | 15.7 (11.21) | 0.5 (0.83) |
| Geometric mean (ng/mL) | 27.5 | 11.7 | 0.2 |
| Geometric CV (%) | 130 | 110 | 320 |
| Range | (0.7, 237.6) | (0.7, 47.6) | (0.0, 3.7) |

**Abbreviations:** CV, coefficient of variation; N, number of observations; PKS, pharmacokinetics set; SD, standard deviation.

## Table S11. Spearman correlation results between viral load reduction and time to sustained resolution of targeted COVID-19 symptoms

| **Days after treatment** | **Statistics** | **SNS812 200 mg group** | **SNS812 100mg group** | **Placebo group** | **Overall** |
| --- | --- | --- | --- | --- | --- |
| Day 1 | Spearman's ρ | -0.20 | 0.08 | -0.07 | -0.10 |
|  | *P*-value | 0.1883 | 0.6004 | 0.6667 | 0.2407 |
| Day 2 | Spearman's ρ | 0.08 | -0.21 | -0.29 | -0.17 |
|  | *P*-value | 0.6060 | 0.1672 | 0.0544 | 0.0465 |
| Day 3 | Spearman's ρ | -0.12 | -0.03 | -0.20 | -0.13 |
|  | *P*-value | 0.4381 | 0.8325 | 0.2026 | 0.1512 |
| Day 4 | Spearman's ρ | -0.21 | -0.34 | -0.31 | -0.33 |
|  | *P*-value | 0.1582 | 0.0294 | 0.0416 | 0.0002 |

**Spearman's ρ: Spearman Correlation Coefficients**

## Table S12. Time to sustained resolution of individual COVID-19 symptoms, by modified intention-to-treat Set (Table S7.1), and by patients with symptom in each group (Table S7.2)

**Table S12.1.** Time to sustained resolution of individual COVID-19 symptoms by the modified intention-to-treat Set

| Modified Intention-to-Treat Set Analysis* | | | | |
| --- | --- | --- | --- | --- |
| Symptom | No. of Patients | Median time (IQR) - day | *P* Value between trial drug and placebo | Adjust *P* Value^#^ between trial drug and placebo |
| Chills or shivers |  |  |  |  |
| SNS812 200 mg group | 45 | 0.3 (0.2–0.7) | **0.003** | **0.008** |
| SNS812 100 mg group | 43 | 0.5 (0.3–1.6) | 0.156 | 0.467 |
| Placebo group | 45 | 0.7 (0.3–1.8) | - | - |
| Loss of taste |  |  |  |  |
| SNS812 200 mg group | 45 | 0.4 (0.2–2.6) | **0.006** | **0.018** |
| SNS812 100 mg group | 43 | 0.5 (0.2–3.4) | 0.034 | 0.102 |
| Placebo group | 45 | 0.5 (0.3–7.8) | - | - |
| Shortness of breath |  |  |  |  |
| SNS812 200 mg group | 45 | 0.3 (0.2–1.2) | **0.007** | **0.021** |
| SNS812 100 mg group | 43 | 0.6 (0.2–2.9) | 0.395 | >0.999 |
| Placebo group | 45 | 0.9 (0.3–2.6) | - | - |
| Stuffy or runny nose |  |  |  |  |
| SNS812 200 mg group | 45 | 5.3 (3.2–7.9) | **0.025** | 0.076 |
| SNS812 100 mg group | 43 | 6.6 (4.7–9.3) | 0.198 | 0.593 |
| Placebo group | 45 | 7.6 (4.6–13.6) | - | - |
| Loss of smell |  |  |  |  |
| SNS812 200 mg group | 45 | 0.5 (0.2–4.3) | **0.028** | 0.083 |
| SNS812 100 mg group | 43 | 0.6 (0.2–4.1) | **0.037** | 0.111 |
| Placebo group | 45 | 1.4 (0.2–8.3) | - | - |
| Nausea |  |  |  |  |
| SNS812 200 mg group | 45 | 0.3 (0.2–0.5) | **0.025** | 0.075 |
| SNS812 100 mg group | 43 | 0.5 (0.2–2.4) | 0.589 | >0.999 |
| Placebo group | 45 | 0.4 (0.2–1.2) | - | - |
| Fever |  |  |  |  |
| SNS812 200 mg group | 45 | 0.7 (0.3–2.0) | 0.075 | 0.224 |
| SNS812 100 mg group | 43 | 0.8 (0.3–1.7) | 0.124 | 0.373 |
| Placebo group | 45 | 1.3 (0.4–2.5) | - | - |
| Sore or dry throat |  |  |  |  |
| SNS812 200 mg group | 45 | 5.3 (3.3–7.2) | 0.076 | 0.228 |
| SNS812 100 mg group | 43 | 5.7 (3.3–7.4) | 0.159 | 0.477 |
| Placebo group | 45 | 6.7 (3.7–9.2) | - | - |
| Diarrhea |  |  |  |  |
| SNS812 200 mg group | 45 | 0.3 (0.2–1.3) | 0.152 | 0.455 |
| SNS812 100 mg group | 43 | 0.3 (0.2–0.6) | 0.563 | >0.999 |
| Placebo group | 45 | 0.3 (0.2–0.5) | - | - |
| Headache |  |  |  |  |
| SNS812 200 mg group | 45 | 1.5 (0.3–2.3) | 0.272 | 0.816 |
| SNS812 100 mg group | 43 | 1.5 (0.4–4.3) | 0.990 | >0.999 |
| Placebo group | 45 | 1.9 (1.1–3.5) | - | - |
| Muscle or body aches |  |  |  |  |
| SNS812 200 mg group | 45 | 1.3 (0.7–2.6) | 0.289 | 0.868 |
| SNS812 100 mg group | 43 | 2.5 (0.7–4.3) | 0.996 | >0.999 |
| Placebo group | 45 | 2.0 (0.9–4.3) | - | - |
| Low energy or tiredness |  |  |  |  |
| SNS812 200 mg group | 45 | 3.0 (1.6–6.7) | 0.445 | >0.999 |
| SNS812 100 mg group | 43 | 4.0 (1.4–6.2) | 0.591 | >0.999 |
| Placebo group | 45 | 4.2 (1.9–8.4) | - | - |
| Cough |  |  |  |  |
| SNS812 200 mg group | 45 | 7.6 (5.3–12.8) | 0.791 | >0.999 |
| SNS812 100 mg group | 43 | 6.4 (4.4–16.6) | 0.643 | >0.999 |
| Placebo group | 45 | 9.3 (5.4–15.4) | - | - |
| Vomiting |  |  |  |  |
| SNS812 200 mg group | 45 | 0.3 (0.2–0.3) | 0.974 | >0.999 |
| SNS812 100 mg group | 43 | 0.3 (0.2–0.4) | 0.116 | 0.347 |
| Placebo group | 45 | 0.2 (0.2–0.3) | - | - |

* In the modified Intention-to-Treat (mITT) population, two patients of the 100 mg group were excluded. One patient withdrew from the trial before receiving any treatment, and the other one was excluded based on the clinical investigator’s assessment that the patient’s pre-existing chronic condition could significantly impact the evaluation of the efficacy and safety of the study drug. As a result, a total of 133 patients were included in the mITT analysis.

^#^ Adjust *P* values were adjusted for multiple comparisons using the Bonferroni correction.

**Table S12.2.** Time to sustained resolution of individual COVID-19 symptoms by patients with symptom in each group

| Patients with Symptom (Subgroup Analysis) | | | | |
| --- | --- | --- | --- | --- |
| Symptom | No. of Patients | Median time (IQR) - day | *P* Value between trial drug and placebo | Adjust *P* Value^#^ between trial drug and placebo |
| Chills or shivers |  |  |  |  |
| SNS812 200 mg group | 26 | 0.6 (0.3–1.5) | **0.008** | **0.023** |
| SNS812 100 mg group | 27 | 1.1 (0.3–1.7) | 0.195 | 0.585 |
| Placebo group | 28 | 1.3 (0.7–2.3) | - | - |
| Loss of taste |  |  |  |  |
| SNS812 200 mg group | 18 | 3.3 (2.3–6.1) | **0.002** | **0.006** |
| SNS812 100 mg group | 20 | 3.4 (2.6–4.9) | **0.002** | **0.005** |
| Placebo group | 21 | 7.8 (3.7–12.) | - | - |
| Shortness of breath |  |  |  |  |
| SNS812 200 mg group | 19 | 1.4 (0.7–2.5) | **0.010** | **0.031** |
| SNS812 100 mg group | 26 | 2.0 (0.7–4.1) | 0.151 | 0.453 |
| Placebo group | 23 | 2.5 (1.7–4.7) | - | - |
| Stuffy or runny nose |  |  |  |  |
| SNS812 200 mg group | 43 | 5.3 (3.3–8.3) | **0.042** | 0.125 |
| SNS812 100 mg group | 42 | 6.7 (4.8–10.0) | 0.234 | 0.702 |
| Placebo group | 45 | 7.6 (4.6–13.6) | - | - |
| Loss of smell |  |  |  |  |
| SNS812 200 mg group | 21 | 4.7 (2.3–6.9) | **0.020** | 0.060 |
| SNS812 100 mg group | 21 | 4.3 (3.3–5.3) | **0.014** | **0.043** |
| Placebo group | 22 | 6.6 (3.3–15.4) | - |  |
| Nausea |  |  |  |  |
| SNS812 200 mg group | 11/11 | 0.9 (0.7–1.3) | 0.169 | 0.507 |
| SNS812 100 mg group | 20/19 | 2.3 (0.9–5.7) | 0.520 | >0.999 |
| Placebo group | 19/19 | 1.2 (0.6–4.6) | - | - |
| Fever |  |  |  |  |
| SNS812 200 mg group | 33 | 1.3 (0.7–2.3) | 0.143 | 0.430 |
| SNS812 100 mg group | 32 | 1.3 (0.6–2.2) | 0.201 | 0.603 |
| Placebo group | 37 | 1.7 (1.1–2.6) | - | - |
| Sore or dry throat |  |  |  |  |
| SNS812 200 mg group | 44 | 5.3 (3.3–7.3) | 0.092 | 0.276 |
| SNS812 100 mg group | 41 | 5.8 (3.4–7.5) | 0.229 | 0.686 |
| Placebo group | 45 | 6.7 (3.7–9.2) |  | - |
| Diarrhea |  |  |  |  |
| SNS812 200 mg group | 15 | 2.7 (1.3–8.7) | 0.549 | >0.999 |
| SNS812 100 mg group | 13 | 2.2 (1.3–4.0) | 0.281 | 0.843 |
| Placebo group | 6 | 2.5 (2.3–6.6) | - | - |
| Headache |  |  |  |  |
| SNS812 200 mg group | 38 | 1.7 (0.9–3.1) | 0.417 | >0.999 |
| SNS812 100 mg group | 35 | 3.4 (0.7–5.0) | 0.600 | >0.999 |
| Placebo group | 39 | 2.1 (1.2–3.7) | - |  |
| Muscle or body aches |  |  |  |  |
| SNS812 200 mg group | 39 | 1.6 (0.8–3.3) | 0.311 | 0.932 |
| SNS812 100 mg group | 36 | 3.1 (1.3–5.7) | 0.837 | >0.999 |
| Placebo group | 38 | 2.2 (1.5–6.7) | - | - |
| Low energy or tiredness |  |  |  |  |
| SNS812 200 mg group | 42 | 3.1 (2.0–7.5) | 0.577 | >0.999 |
| SNS812 100 mg group | 39 | 4.3 (1.9–6.4) | 0.847 | >0.999 |
| Placebo group | 44 | 4.2 (1.9–8.4) | - | - |
| Cough |  |  |  |  |
| SNS812 200 mg group | 43 | 7.6 (5.4–12.8) | 0.878 | >0.999 |
| SNS812 100 mg group | 43 | 6.4 (4.4–16.6) | 0.719 | >0.999 |
| Placebo group | 44 | 9.3 (5.4–15.6) | - | - |
| Vomiting |  |  |  |  |
| SNS812 200 mg group | 1 | 0.5 (0.5–0.5) | - | - |
| SNS812 100 mg group | 7 | 0.8 (0.3–2.4) | - | - |
| Placebo group | 0 | - |  | - |

^#^ Adjust *P* values were adjusted for multiple comparisons using the Bonferroni correction.

## Table S13. Time to sustained alleviation of individual COVID-19 symptoms, by modified intention-to-treat Set (Table S8.1), and by patients with symptom in each group (Table S8.2)

**Table S13.1.** Time to sustained alleviation of individual COVID-19 symptoms by the modified intention-to-treat Set

| Modified Intention-to-Treat Set Analysis* | | | | |
| --- | --- | --- | --- | --- |
| Symptom | No. of Patients | Median time (IQR) - day | *P* Value between trial drug and placebo | Adjust *P* Value^#^ between trial drug and placebo |
| Chills or shivers |  |  |  |  |
| SNS812 200 mg group | 45 | 0.3 (0.2–0.5) | **<0.001** | **0.001** |
| SNS812 100 mg group | 43 | 0.4 (0.2–1.0) | 0.066 | 0.197 |
| Placebo group | 45 | 0.5 (0.3–1.7) | - | - |
| Loss of taste |  |  |  |  |
| SNS812 200 mg group | 45 | 0.4 (0.2–2.5) | **0.008** | **0.023** |
| SNS812 100 mg group | 43 | 0.5 (0.2–3.4) | **0.041** | 0.123 |
| Placebo group | 45 | 0.5 (0.3–6.6) | - | - |
| Shortness of breath |  |  |  |  |
| SNS812 200 mg group | 45 | 0.3 (0.2–0.9) | **0.014** | **0.041** |
| SNS812 100 mg group | 43 | 0.5 (0.2–1.6) | 0.243 | 0.729 |
| Placebo group | 45 | 0.5 (0.3–2.5) | - | - |
| Stuffy or runny nose |  |  |  |  |
| SNS812 200 mg group | 45 | 1.7 (0.7–3.6) | 0.236 | 0.707 |
| SNS812 100 mg group | 43 | 3.4 (1.0–6.5) | 0.941 | >0.999 |
| Placebo group | 45 | 3.3 (1.2–7.3) | - | - |
| Loss of smell |  |  |  |  |
| SNS812 200 mg group | 45 | 0.5 (0.2–3.6) | **0.050** | 0.155 |
| SNS812 100 mg group | 43 | 0.6 (0.2–4.1) | 0.059 | 0.176 |
| Placebo group | 45 | 1.2 (0.2–6.6) | - | - |
| Nausea |  |  |  |  |
| SNS812 200 mg group | 45 | 0.3 (0.2–0.5) | 0.072 | 0.216 |
| SNS812 100 mg group | 43 | 0.4 (0.2–2.3) | 0.283 | 0.850 |
| Placebo group | 45 | 0.3 (0.2–1.0) | - | - |
| Fever |  |  |  |  |
| SNS812 200 mg group | 45 | 0.3 (0.2–0.9) | 0.073 | 0.128 |
| SNS812 100 mg group | 43 | 0.4 (0.2–1.4) | 0.321 | 0.695 |
| Placebo group | 45 | 0.7 (0.3–1.7) | - | - |
| Sore or dry throat |  |  |  |  |
| SNS812 200 mg group | 45 | 2.3 (1.3–3.8) | 0.239 | 0.718 |
| SNS812 100 mg group | 43 | 3.2 (1.3–5.1) | 0.441 | >0.999 |
| Placebo group | 45 | 3.0 (1.5–6.6) | - | - |
| Diarrhea |  |  |  |  |
| SNS812 200 mg group | 45 | 0.3 (0.2–0.8) | 0.205 | 0.616 |
| SNS812 100 mg group | 43 | 0.3 (0.2–0.5) | 0.732 | >0.999 |
| Placebo group | 45 | 0.3 (0.2–0.5) | - | - |
| Headache |  |  |  |  |
| SNS812 200 mg group | 45 | 0.9 (0.3–1.9) | 0.410 | >0.999 |
| SNS812 100 mg group | 43 | 0.7 (0.3–2.9) | 0.417 | >0.999 |
| Placebo group | 45 | 1.3 (0.7–2.3) | - | - |
| Muscle or body aches |  |  |  |  |
| SNS812 200 mg group | 45 | 1.0 (0.3–1.6) | 0.260 | 0.779 |
| SNS812 100 mg group | 43 | 1.2 (0.4–3.9) | 0.314 | 0.941 |
| Placebo group | 45 | 1.3 (0.3–2.6) | - | - |
| Low energy or tiredness |  |  |  |  |
| SNS812 200 mg group | 45 | 1.5 (0.3–2.7) | 0.596 | >0.999 |
| SNS812 100 mg group | 43 | 1.8 (0.5–2.9) | 0.714 | >0.999 |
| Placebo group | 45 | 1.7 (1.1–3.3) | - | - |
| Cough |  |  |  |  |
| SNS812 200 mg group | 45 | 4.6 (1.7–7.6) | 0.292 | 0.877 |
| SNS812 100 mg group | 43 | 2.9 (1.3–5.6) | **0.013** | 0.040 |
| Placebo group | 45 | 4.5 (1.8–12.5) | - | - |
| Vomiting |  |  |  |  |
| SNS812 200 mg group | 45 | 0.3 (0.2–0.3) | 0.974 | >0.999 |
| SNS812 100 mg group | 43 | 0.3 (0.2–0.4) | 0.116 | 0.347 |
| Placebo group | 45 | 0.2 (0.2–0.3) | - | - |

* In the modified Intention-to-Treat (mITT) population, two patients of the 100 mg group were excluded. One patient withdrew from the trial before receiving any treatment, and the other one was excluded based on the clinical investigator’s assessment that the patient’s pre-existing chronic condition could significantly impact the evaluation of the efficacy and safety of the study drug. As a result, a total of 133 patients were included in the mITT analysis.

^#^ Adjust *P* values were adjusted for multiple comparisons using the Bonferroni correction.

**Table S13.2.** Time to sustained alleviation of individual COVID-19 symptoms by patients with symptom in each group

| Patients with Symptom (Subgroup Analysis) | | | | |
| --- | --- | --- | --- | --- |
| Symptom | No. of Patients | Median time (IQR) - day | *P* Value between trial drug and placebo | Adjust *P* Value^#^ between trial drug and placebo |
| Chills or shivers |  |  |  |  |
| SNS812 200 mg group | 26 | 0.3 (0.2–0.9) | **0.001** | **0.004** |
| SNS812 100 mg group | 27 | 0.8 (0.3–1.6) | 0.089 | 0.268 |
| Placebo group | 28 | 1.1 (0.5–2.3) | - | - |
| Loss of taste |  |  |  |  |
| SNS812 200 mg group | 18 | 3.2 (2.2–6.1) | **0.003** | **0.009** |
| SNS812 100 mg group | 20 | 3.4 (2.4–4.9) | **0.003** | **0.008** |
| Placebo group | 21 | 6.6 (3.7–12.3) | - | - |
| Shortness of breath |  |  |  |  |
| SNS812 200 mg group | 19 | 1.3 (0.4–2.5) | **0.025** | 0.074 |
| SNS812 100 mg group | 26 | 1.3 (0.4–3.2) | 0.073 | 0.220 |
| Placebo group | 23 | 2.3 (1.7–3.8) | - | - |
| Stuffy or runny nose |  |  |  |  |
| SNS812 200 mg group | 43 | 1.7 (0.7–3.6) | 0.327 | 0.981 |
| SNS812 100 mg group | 42 | 3.4 (1.3–6.5) | 0.854 | >0.999 |
| Placebo group | 45 | 3.3 (1.2–7.3) | - | - |
| Loss of smell |  |  |  |  |
| SNS812 200 mg group | 21 | 4.3 (1.7–6.9) | 0.058 | 0.173 |
| SNS812 100 mg group | 21 | 4.2 (3.3–5.2) | 0.032 | 0.096 |
| Placebo group | 22 | 5.3 (2.2–11.3) | - |  |
| Nausea |  |  |  |  |
| SNS812 200 mg group | 11 | 0.8 (0.3–1.3) | 0.458 | >0.999 |
| SNS812 100 mg group | 20 | 2.3 (0.7–5.7) | 0.177 | 0.532 |
| Placebo group | 19 | 1.2 (0.6–2.6) | - | - |
| Fever |  |  |  |  |
| SNS812 200 mg group | 33 | 0.6 (0.3–1.3) | 0.073 | 0.219 |
| SNS812 100 mg group | 32 | 0.7 (0.3–1.8) | 0.321 | 0.962 |
| Placebo group | 37 | 1.1 (0.4–2.0) | - | - |
| Sore or dry throat |  |  |  |  |
| SNS812 200 mg group | 44 | 2.3 (1.3–4.0) | 0.277 | 0.832 |
| SNS812 100 mg group | 41 | 3.2 (1.8–5.7) | 0.573 | >0.999 |
| Placebo group | 45 | 3.0 (1.5–6.6) |  | - |
| Diarrhea |  |  |  |  |
| SNS812 200 mg group | 15 | 2.7 (0.8–8.7) | 0.560 | >0.999 |
| SNS812 100 mg group | 13 | 2.2 (0.2–4.0) | 0.294 | 0.881 |
| Placebo group | 6 | 2.5 (2.3–6.6) | - | - |
| Headache |  |  |  |  |
| SNS812 200 mg group | 38 | 1.4 (0.5–2.1) | 0.629 | >0.999 |
| SNS812 100 mg group | 35 | 1.3 (0.5–3.3) | 0.728 | >0.999 |
| Placebo group | 39 | 1.5 (0.9–2.5) | - |  |
| Muscle or body aches |  |  |  |  |
| SNS812 200 mg group | 39 | 1.2 (0.7–2.0) | 0.289 | 0.868 |
| SNS812 100 mg group | 36 | 2.1 (0.7–4.2) | 0.193 | 0.580 |
| Placebo group | 38 | 1.5 (0.5–2.7) | - | - |
| Low energy or tiredness |  |  |  |  |
| SNS812 200 mg group | 42 | 1.6 (0.4–3.0) | 0.748 | >0.999 |
| SNS812 100 mg group | 39 | 2.2 (0.7–3.5) | 0.488 | >0.999 |
| Placebo group | 44 | 1.7 (1.2–3.3) | - | - |
| Cough |  |  |  |  |
| SNS812 200 mg group | 43 | 4.7 (1.7–7.6) | 0.337 | >0.999 |
| SNS812 100 mg group | 43 | 2.9 (1.3–5.6) | 0.009 | 0.027 |
| Placebo group | 44 | 4.5 (1.8–12.5) | - | - |
| Vomiting |  |  |  |  |
| SNS812 200 mg group | 1 | 0.5 (0.5–0.5) | - | - |
| SNS812 100 mg group | 7 | 0.8 (0.3–2.4) | - | - |
| Placebo group | 0 | - |  | - |

^#^ Adjust *P* values were adjusted for multiple comparisons using the Bonferroni correction.

| **Patient** | **Sex** | **Age** | **Notable Activity Prior to CK Elevation** |
| --- | --- | --- | --- |
| S010 | Male | 30 | Played volleyball the night before |
| S046 | Female | 46 | Weight training at the gym and power walking 15,000 steps |
| S086 | Male | 32 | Weight training at the gym |
| S119 | Male | 46 | Weight training at the gym |
| S152 | Female | 23 | Swimming |
| S013 | Male | 34 | Weight training at the gym |
| S140 | Female | 31 | Weight training at the gym and jogging |

## Figure S1. Timing and peak value of creatine kinase (CK) elevation in seven participants

The figure displays CK levels over time in participants who experienced CK elevation. None of these participants had any known comorbid medical conditions. The table summarizes findings from physician-conducted interviews regarding physical activities that occurred prior to the detection of abnormal CK values. The green arrow in the figure indicates the time of viral clearance, as confirmed by a COVID-19 antigen test.

**Figure S2. Improvement of individual COVID-19 symptoms following treatment with 200 mg SNS812 (mITT: participants with the symptom)**

Improvement of individual COVID-19–related symptoms among participants who presented with the respective symptoms at baseline, comparing the 200 mg SNS812 group with the placebo group. Bar graphs display the percentage change in the median time to symptom resolution (A) or symptom alleviation (B) in the 200 mg treatment group relative to placebo. Black asterisks indicate symptoms with nominal P values < 0.05 prior to Bonferroni correction, whereas red asterisks denote symptoms that remained statistically significant after Bonferroni correction (P < 0.05). Vomiting was excluded from the analysis due to the limited number of evaluable cases.

## Figure S3. Kaplan–Meier analysis of time to sustained resolution and alleviation of shortness of breath (mITT population)

Kaplan–Meier analysis was performed to evaluate the efficacy of SNS812 in the mITT population with a specific focus on the symptom of shortness of breath. Kaplan–Meier curves for TTSR of shortness of breath are shown in the left panel (3A), and those for TTSA are shown in the right panel (3B). The 200 mg SNS812 group is indicated by red dashed lines, the 100 mg SNS812 group by blue, and the placebo group by black.

## Figure S4. Kaplan–Meier analysis of time to sustained resolution and alleviation of chills and shivers (mITT population)

Kaplan–Meier analysis was performed to evaluate the efficacy of SNS812 in the mITT population with a specific focus on the symptom of chills and shivers. Kaplan–Meier curves for TTSR of chills and shivers are shown in the left panel (3A), and those for TTSA are shown in the right panel (3B). The 200 mg SNS812 group is indicated by red dashed lines, the 100 mg SNS812 group by blue, and the placebo group by black.

## Figure S5. Kaplan–Meier analysis of time to sustained resolution and alleviation of loss of taste and smell (mITT population)

## Kaplan–Meier analysis was performed to evaluate the efficacy of SNS812 in the mITT population with a specific focus on the symptoms of loss of taste and loss of smell. Kaplan–Meier curves for TTSR and TTSA of loss of taste are shown in panels A and B, respectively, while those for loss of smell are shown in panels C and D, respectively. The 200 mg SNS812 group is indicated by red dashed lines, the 100 mg SNS812 group by blue dashed lines, and the placebo group by black lines.

# Appendix 4 Summary of Safety Evaluation from CSR

## Overall Summary of Safety Evaluation

The summary report of the safety evaluation is adopted from the CSR (Section 14). All safety analyses were conducted using the SS (Safety Set). Safety evaluations included the incidence of TEAEs, AEs, TRAEs, SAEs, changes from baseline in laboratory values, vital signs, and other safety examination parameters.

## 1.1. AEs

### 1.1.1.  **Brief Summary of the AEs**

TEAEs were defined as AEs that began on or after the first treatment. A summary of TEAEs by participant is provided in **Table 1**, while a summary by event can be found in **Table 2**.

A total of 158 TEAEs were reported in 72 (53.73%) participants, with 2 SAEs occurring in 1 (0.75%) participant. No participants had TEAEs that led to treatment withdrawal.

#### 1.1.1.1. **Summary of the TEAEs**

In the 100-mg group, 24 (54.55%) participants reported a total of 57 TEAEs; in the 200-mg group, 27 (60%) participants reported 50 TEAEs; and in the placebo group, 21 (46.67%) participants reported 51 TEAEs. In the 200-mg group, among the 50 TEAEs, 35 (70%) were mild, 12 (24%) were moderate, and 3 (6%) were severe. In the 100-mg group, of the 57 TEAEs, 38 (66.67%) were mild, 14 (24.56%) were moderate, and 5 (8.77%) were severe. In the placebo group, 38 (74.51%) of the 51 TEAEs were mild and 13 (25.49%) were moderate. Regarding the relationship of TEAEs to the study treatment, 136 (86.08%) TEAEs were assessed as unrelated, while 22 (13.92%) were deemed unlikely related. For 119 (75.32%) TEAEs, no action was taken, and 39 (24.68%) were not applicable to the actions taken with the study treatment. Among the 158 TEAEs, 2 (1.27%) were still recovering, 18 (11.39%) had not resolved, and 138 (87.34%) had resolved by the time the database was locked. Only one participant in the 100-mg group experienced two SAEs that were unrelated to the study treatment.

- **Table 1: Summary of Treatment-Emergent Adverse Events (By subjects; SS)**

| **TEAEs** | **SNS812**  **200 mg**  **(N = 45)** | **SNS812**  **100 mg (N = 44)** | **Placebo**  **(N = 45)** | **Total (N = 134)** |
| --- | --- | --- | --- | --- |
| **Summary of AEs** | | | | |
| Number of participants with TEAEs | 27 (60%) | 24 (54.55%) | 21 (46.67%) | 72 (53.73%) |
| Number of participants with treatment withdrawal due to TEAEs | 0 | 0 | 0 | 0 |
| Number of participants with SAEs | 0 | 1 (2.27%) | 0 | 1 (0.75%) |
| **Severity of TEAEs*** | | | | |
| Mild | 13 (28.89%) | 15 (34.09%) | 12 (26.67%) | 40 (29.85%) |
| Moderate | 11 (24.44%) | 6 (13.64%) | 9 (20%) | 26 (19.40%) |
| Severe | 3 (6.67%) | 3 (6.82%) | 0 | 6 (4.48%) |
| Life-threatening | 0 | 0 | 0 | 0 |
| Death | 0 | 0 | 0 | 0 |
| **Relationship of TEAEs to study treatment*** | | | | |
| Unrelated | 22 (48.89%) | 19 (43.18%) | 15 (33.33%) | 56 (41.79%) |
| Unlikely | 5 (11.11%) | 5 (11.36%) | 6 (13.33%) | 16 (11.94%) |
| Possibly | 0 | 0 | 0 | 0 |
| Probably | 0 | 0 | 0 | 0 |
| Definitely | 0 | 0 | 0 | 0 |
| Abbreviations: AEs, adverse events; TEAEs, treatment-emergent adverse events; SAEs, serious adverse events; SS, safety set.  Percentage of patients with event: Number of patients with events/Total number of subjects.  *Calculated based on the highest level of severity or relationship of each participant. | | | | |

- **Table 2: Summary of Treatment-Emergent Adverse Events (By events; SS)**

| **TEAEs** | **SNS812**  **200 mg**  **(N = 45)** | **SNS812**  **100 mg (N = 44)** | **Placebo**  **(N = 45)** | **Total (N = 134)** |
| --- | --- | --- | --- | --- |
| **Summary of AEs** | | | | |
| Number of TEAEs | 50 | 57 | 51 | 158 |
| Number of SAEs | 0 | 2 | 0 | 2 |
| **Severity of TEAEs** | | | | |
| Mild | 35 (70%) | 38 (66.67%) | 38 (74.51%) | 111 (70.25%) |
| Moderate | 12 (24%) | 14 (24.56%) | 13 (25.49%) | 39 (24.68%) |
| Severe | 3 (6%) | 5 (8.77%) | 0 | 8 (5.06%) |
| Life-threatening | 0 | 0 | 0 | 0 |
| Death | 0 | 0 | 0 | 0 |
| **Relationship of TEAEs to study treatment** | | | | |
| Unrelated | 45 (90%) | 51 (89.47%) | 40 (78.43%) | 136 (86.08%) |
| Unlikely | 5 (10%) | 6 (10.53%) | 11 (21.57%) | 22 (13.92%) |
| Possibly | 0 | 0 | 0 | 0 |
| Probably | 0 | 0 | 0 | 0 |
| Definitely | 0 | 0 | 0 | 0 |
| **Action taken to study treatment** | | | | |
| Dose unchanged | 28 (56%) | 48 (84.21%) | 43 (84.31%) | 119 (75.32%) |
| Not applicable | 22 (44%) | 9 (15.79%) | 8 (15.69%) | 39 (24.68%) |
| **TEAEs outcome** | | | | |
| Resolved | 42 (84%) | 50 (87.72%) | 46 (90.20%) | 138 (87.34%) |
| Recovering | 1 (2%) | 1 (1.75%) | 0 | 2 (1.27%) |
| Not resolved | 7 (14%) | 6 (10.53%) | 5 (9.80%) | 18 (11.39%) |
| Abbreviations: AEs, adverse events; N, number of participants TEAEs, treatment-emergent adverse events; SAEs, serious adverse events; SS, safety set.  Percentage of event: Number of events/Total TEAEs | | | | |

### 1.1.2.  Display of the TEAEs

The AEs were coded using the MedDRA^®^ coding system (version 26.1) and categorized by SOC and PT (**Table 3**). Among the 158 TEAEs reported in 72 participants, the most frequent SOCs were nervous system disorders (38 participants, 28.36%), followed by investigations (17 participants, 12.69%) and gastrointestinal disorders (16 participants, 11.94%). The most common PTs included anosmia (20 participants, 14.93%), dysgeusia (12 participants, 8.96%), and diarrhea (11 participants, 8.21%). The incidence of TEAEs by PT was generally similar across the three study groups, except for increased blood creatine phosphokinase (CPK), which occurred in 4 (4.5%) participants in the 200-mg group, 2 (8.9%) in the 100-mg group, and none in the placebo group. The elevated CPK levels in these participants were attributed to exercise and were thus considered unrelated to treatment. For more details on the increased blood CPK events, refer to Section 1.3.2. Most TEAEs were associated with the participants’ underlying conditions or the nature of COVID-19 infection.

- **Table 3: Summary of Treatment-Emergent Adverse Events by SOC and PT by Participant (SS)**

| **SOC/PT of TEAEs** | **SNS812**  **200 mg**  **(N = 45)** | **SNS812**  **100 mg (N = 44)** | **Placebo**  **(N = 45)** | **Total**  **(N = 134)** | ***p*-value^#^** |
| --- | --- | --- | --- | --- | --- |
| Cardiac disorders | 0 | 1 (2.27%) | 0 | 1 (0.75%) | 0.328 |
| Palpitations | 0 | 1 (2.27%) | 0 | 1 (0.75%) | 0.328 |
| Eye disorders | 0 | 2 (4.55%) | 0 | 2 (1.49%) | 0.106 |
| Dry eye | 0 | 1 (2.27%) | 0 | 1 (0.75%) | 0.328 |
| Photophobia | 0 | 1 (2.27%) | 0 | 1 (0.75%) | 0.328 |
| Gastrointestinal disorders | 2 (4.44%) | 8 (18.18%) | 6 (13.33%) | 16 (11.94%) | 0.096 |
| Abdominal pain | 1 (2.22%) | 0 | 0 | 1 (0.75%) | >0.999 |
| Diarrhoea | 2 (4.44%) | 5 (11.36%) | 4 (8.89%) | 11 (8.21%) | 0.460 |
| Gastrointestinal pain | 0 | 0 | 1 (2.22%) | 1 (0.75%) | >0.999 |
| Haemorrhoids | 0 | 0 | 1 (2.22%) | 1 (0.75%) | >0.999 |
| Nausea | 1 (2.22%) | 3 (6.82%) | 0 | 4 (2.99%) | 0.127 |
| Vomiting | 0 | 2 (4.55%) | 1 (2.22%) | 3 (2.24%) | 0.323 |
| General disorders and administration site conditions | 2 (4.44%) | 2 (4.55%) | 2 (4.44%) | 6 (4.48%) | >0.999 |
| Chills | 0 | 1 (2.27%) | 0 | 1 (0.75%) | 0.328 |
| Influenza-like illness | 1 (2.22%) | 0 | 0 | 1 (0.75%) | >0.999 |
| Malaise | 0 | 1 (2.27%) | 2 (4.44%) | 3 (2.24%) | 0.546 |
| Pyrexia | 1 (2.22%) | 1 (2.27%) | 0 | 2 (1.49%) | 0.773 |
| Infections and infestations | 6 (13.33%) | 3 (6.82%) | 4 (8.89%) | 13 (9.70%) | 0.669 |
| Acute sinusitis | 1 (2.22%) | 0 | 1 (2.22%) | 2 (1.49%) | >0.999 |
| Bronchitis | 0 | 0 | 1 (2.22%) | 1 (0.75%) | >0.999 |
| Conjunctivitis | 1 (2.22%) | 0 | 0 | 1 (0.75%) | >0.999 |
| Pharyngitis | 1 (2.22%) | 1 (2.27%) | 0 | 2 (1.49%) | 0.773 |
| Rash pustular | 1 (2.22%) | 0 | 0 | 1 (0.75%) | >0.999 |
| Tinea pedis | 0 | 0 | 1 (2.22%) | 1 (0.75%) | >0.999 |
| Tonsillitis | 0 | 1 (2.27%) | 0 | 1 (0.75%) | 0.328 |
| Upper respiratory tract infection | 2 (4.44%) | 1 (2.27%) | 2 (4.44%) | 5 (3.73%) | >0.999 |
| Injury, poisoning, and procedural complications | 1 (2.22%) | 0 | 0 | 1 (0.75%) | >0.999 |
| Epicondylitis | 1 (2.22%) | 0 | 0 | 1 (0.75%) | >0.999 |
| Investigations | 8 (17.78%) | 5 (11.36%) | 4 (8.89%) | 17 (12.69%) | 0.468 |
| Alanine aminotransferase increased | 0 | 2 (4.55%) | 0 | 2 (1.49%) | 0.106 |
| Aspartate aminotransferase increased | 0 | 2 (4.55%) | 0 | 2 (1.49%) | 0.106 |
| Blood bilirubin increased | 1 (2.22%) | 0 | 1 (2.22%) | 2 (1.49%) | >0.999 |
| Blood creatine phosphokinase increased | 4 (8.89%) | 2 (4.55%) | 0 | 6 (4.48%) | 0.124 |
| Blood creatinine increased | 1 (2.22%) | 0 | 1 (2.22%) | 2 (1.49%) | >0.999 |
| Neutrophil count decreased | 0 | 2 (4.55%) | 2 (4.44%) | 4 (2.99%) | 0.469 |
| Platelet count decreased | 1 (2.22%) | 0 | 0 | 1 (0.75%) | >0.999 |
| White blood cell count decreased | 3 (6.67%) | 1 (2.27%) | 2 (4.44%) | 6 (4.48%) | 0.871 |
| Musculoskeletal and connective tissue disorders | 2 (4.44%) | 1 (2.27%) | 1 (2.22%) | 4 (2.99%) | >0.999 |
| Back pain | 1 (2.22%) | 0 | 0 | 1 (0.75%) | >0.999 |
| Myalgia | 0 | 1 (2.27%) | 1 (2.22%) | 2 (1.49%) | 0.773 |
| Myositis | 1 (2.22%) | 0 | 0 | 1 (0.75%) | >0.999 |
| Neoplasms benign, malignant, and unspecified (incl cysts and polyps) | 1 (2.22%) | 0 | 0 | 1 (0.75%) | >0.999 |
| Fibroadenoma of breast | 1 (2.22%) | 0 | 0 | 1 (0.75%) | >0.999 |
| Nervous system disorders | 10 (22.22%) | 13 (29.55%) | 15 (33.33%) | 38 (28.36%) | 0.503 |
| Ageusia | 2 (4.44%) | 2 (4.55%) | 3 (6.67%) | 7 (5.22%) | >0.999 |
| Anosmia | 3 (6.67%) | 7 (15.91%) | 10 (22.22%) | 20 (14.93%) | 0.120 |
| Dizziness | 3 (6.67%) | 1 (2.27%) | 3 (6.67%) | 7 (5.22%) | 0.699 |
| Dysgeusia | 2 (4.44%) | 5 (11.36%) | 5 (11.11%) | 12 (8.96%) | 0.453 |
| Headache | 3 (6.67%) | 4 (9.09%) | 1 (2.22%) | 8 (5.97%) | 0.358 |
| Respiratory, thoracic and mediastinal disorders | 7 (15.56%) | 3 (6.82%) | 3 (6.67%) | 13 (9.70%) | 0.298 |
| Cough | 1 (2.22%) | 0 | 0 | 1 (0.75%) | >0.999 |
| Dyspnoea | 1 (2.22%) | 0 | 2 (4.44%) | 3 (2.24%) | 0.773 |
| Nasal congestion | 1 (2.22%) | 1 (2.27%) | 0 | 2 (1.49%) | 0.773 |
| Nasal obstruction | 2 (4.44%) | 1 (2.27%) | 1 (2.22%) | 4 (2.99%) | >0.999 |
| Oropharyngeal pain | 2 (4.44%) | 1 (2.27%) | 1 (2.22%) | 4 (2.99%) | >0.999 |
| Rhinorrhoea | 1 (2.22%) | 0 | 2 (4.44%) | 3 (2.24%) | 0.773 |
| Skin and subcutaneous tissue disorders | 0 | 3 (6.82%) | 2 (4.44%) | 5 (3.73%) | 0.205 |
| Hyperhidrosis | 0 | 1 (2.27%) | 0 | 1 (0.75%) | 0.328 |
| Pruritus | 0 | 1 (2.27%) | 0 | 1 (0.75%) | 0.328 |
| Purpura | 0 | 0 | 1 (2.22%) | 1 (0.75%) | >0.999 |
| Rash maculo-papular | 0 | 0 | 1 (2.22%) | 1 (0.75%) | >0.999 |
| Urticaria | 0 | 1 (2.27%) | 0 | 1 (0.75%) | 0.328 |
| Abbreviations: N, number of participants; PT, preferred term; SOC, system organ class; SS, safety set; TEAEs, treatment-emergent adverse events.  ^#^: *p*-value was calculated by Fisher's exact test.  *: Statistically significant | | | | | |

### 1.1.3.  Brief Summary of the TRAEs

TRAEs were defined as AEs that occurred in participants and were assessed as definitely, probably, or possibly related to the study treatment. No participants experienced TRAEs.

## 1.2.  Deaths, Other SAEs, and Other Significant AEs

### 1.2.1.  Listing of Deaths, Other SAEs, and Other Significant AEs

One participant was reported with two SAEs, and there were no deaths reported during the study.

### 1.2.2.  Narrative of Other SAEs

Subject S140, a 31-year-old female, was enrolled in the study on June 7, 2024. She received 100 mg of IP once daily from June 7 to June 13, 2024.

The participant had a medical history of acute hepatitis in July 2019. A blood examination on the screening day (June 7, 2024) revealed elevated liver function (SGOT, 138 U/L; SGPT, 193 U/L). After completing her treatment, the participant was hospitalized during the follow-up period (from FU2 to FU3) for 2 days (from June 25 to June 27, 2024) due to abnormal liver function (elevated SGOT and SGPT levels). Following her discharge, the participant was closely monitored by the study team until the end of the study (July 29, 2024), during which safety assessments were conducted per protocol, and no additional liver function-related SAEs were reported or identified. The results indicated gradual improvement, nearing normal levels, and no signs of liver cirrhosis were observed during the study period.

Details of SGOT and SGPT are provided below:

|  | **07-Jun** | **08-Jun** | **10-Jun** | **13-Jun** | **15-Jun** | **20-Jun** | **25-Jun** | **04-Jul** | **29-Jul** |
| --- | --- | --- | --- | --- | --- | --- | --- | --- | --- |
| SGOT (U/L) | 138 | 127 | 86 | 200 | 231 | 394 | 241 | 66 | 44 |

|  | **07-Jun** | **08-Jun** | **10-Jun** | **13-Jun** | **15-Jun** | **20-Jun** | **25-Jun** | **04-Jul** | **29-Jul** |
| --- | --- | --- | --- | --- | --- | --- | --- | --- | --- |
| SGPT (U/L) | 193 | 211 | 165 | 288 | 377 | 655 | 621 | 154 | 61 |

During these two incidents, the participant was treated with silymarin and Stronger Neo-Minophagen C (glycyrrhizinate monoammonium). Additionally, due to the participant’s extensive gastrointestinal history, it was noted that she had experienced a similar situation previously without a definitive diagnosis. As a result, the investigator deemed the events unrelated to the study treatment.

## 1.3.  Clinical Laboratory Evaluation

### 1.3.1. Hematology

**Table 4** summarizes CS abnormalities in hematology.

Most CS abnormalities observed in participants were associated with underlying diseases or COVID-19. Participant S096 in the 100-mg group showed a decrease in WBC and neutrophils on Day 4, but these values returned to normal in subsequent visits. Participant S131, also in the 100-mg group, experienced a neutrophil decrease on Day 4, with values returning to normal in later visits.

Participant S003 in the 200-mg group had a WBC decrease on Day 4, which normalized in later visits. Participant S070 in the 200-mg group showed a WBC decrease on Days 7 and 9 and a platelet decrease on Day 9, with values returning to normal afterward. Participant S081 in the 200-mg group experienced a WBC decrease on Days 2 and 4, which also normalized in subsequent visits.

Other participants in the 100-mg and 200-mg groups did not exhibit CS abnormalities during the study, and no identifiable trends related to the IP were noted in hematology.

- **Table 4: Abnormality with Clinical Significance in Hematology**

| **Group** | **Participant** | **Visit** | **Item** | **Value** | **Unit** | **Reason** |
| --- | --- | --- | --- | --- | --- | --- |
| **SNS812**  **200 mg** | 001-S003 (male) | Screening | Lymphocytes | 13.4 | % | COVID-19 related |
|  |  |  | Monocytes | 15.9 | % | COVID-19 related |
|  |  | Day 2 | Monocytes | 16.6 | % | COVID-19 related |
|  |  | Day 4 | WBC | 2.75 | 10^3^/μL | AE: WBC count decreased |
|  | 001-S070 (male) | Day 7/EOT | WBC | 3.02 | 10^3^/μL | AE: WBC count decreased |
|  |  | Day 9 | WBC | 2.75 | 10^3^/μL | AE: WBC count decreased |
|  |  |  | Platelets | 135 | 10^3^/μL | AE: Platelet count decreased |
|  | 001-S081 (male) | Screening | Hemoglobin | 10.9 | g/dL | Thalassaemia |
|  |  |  | Hematocrit | 33.8 | % | Thalassaemia |
|  |  | Day 2 | Hemoglobin | 10.9 | g/dL | Thalassaemia |
|  |  |  | Hematocrit | 34.1 | % | Thalassaemia |
|  |  |  | WBC | 3.13 | 10^3^/μL | AE: WBC count decreased |
|  |  | Day 4 | Hemoglobin | 10.7 | g/dL | Thalassaemia |
|  |  |  | Hematocrit | 34.4 | % | Thalassaemia |
|  |  |  | WBC | 3.10 | 10^3^/μL | AE: WBC count decreased |
|  |  | Day 7/EOT | Hemoglobin | 10.4 | g/dL | Thalassaemia |
|  |  |  | Hematocrit | 33.2 | % | Thalassaemia |
|  |  | Day 9 | Hemoglobin | 10.0 | g/dL | Thalassaemia |
|  |  |  | Hematocrit | 32.1 | % | Thalassaemia |
|  |  | Day 14 | Hemoglobin | 10.2 | g/dL | Thalassaemia |
|  |  |  | Hematocrit | 32.3 | % | Thalassaemia |
|  |  | Day 28 | Hemoglobin | 10.0 | g/dL | Thalassaemia |
|  |  |  | Hematocrit | 31.7 | % | Thalassaemia |
|  |  | Day 60 | Hemoglobin | 10.1 | g/dL | Thalassaemia |
|  |  |  | Hematocrit | 32.4 | % | Thalassaemia |
|  | 007-S152 (female) | Day 28 | Hemoglobin | 11.3 | g/ dL | Menstruation |
|  |  |  | Hematocrit | 36.5 | % | Menstruation |
| **SNS812**  **100 mg** | 001-S028 (female) | Screening | Hemoglobin | 10.7 | g/dL | Microcytic anaemia |
|  |  | Screening | RBC | 5.8 | 10^6^/μL | Microcytic anaemia |
|  |  | Day 2 | RBC | 6.42 | 10^6^/μL | Microcytic anaemia |
|  |  | Day 9 | RBC | 6.05 | 10^6^/μL | Microcytic anaemia |
|  |  | Day 14 | RBC | 5.82 | 10^6^/μL | Microcytic anaemia |
|  |  | Day 28 | RBC | 5.92 | 10^6^/μL | Microcytic anaemia |
|  |  | Day 60 | RBC | 5.96 | 10^6^/μL | Microcytic anaemia |
|  | 005-S047 (female) | Day 7/EOT | WBC | 17.32 | 10^3^/μL | COVID-19 related |
|  |  |  | Neutrophils | 91.4 | % | COVID-19 related |
|  |  | Day 14 | WBC | 11.40 | 10^3^/μL | COVID-19 related |
|  |  |  | Neutrophils | 77.4 | % | COVID-19 related |
|  |  |  | Lymphocytes | 15.2 | % | COVID-19 related |
|  | 001-S096 (male) | Day 4 | WBC | 2.91 | 10^3^/μL | AE: WBC count decreased |
|  |  |  | Neutrophils | 33 | % | AE: Neutrophil count decreased |
|  | 001-S131 (female) | Day 4 | Neutrophils | 35 | % | AE: Neutrophil count decreased |
|  | 007-S153 (female) | Day 4 | Neutrophils | 36.5 | % | Covid-19 related |
|  |  | Day 4 | Lymphocytes | 53.6 | % | Covid-19 related |
| **Placebo** | 001-S040 (male) | Screening | RBC | 6.73 | 10^6^/μL | Thalassaemia |
|  |  | Day 2 | RBC | 6.38 | 10^6^/μL | Thalassaemia |
|  |  | Day 4 | Hemoglobin | 11.6 | g/ dL | Thalassaemia |
|  |  |  | Hematocrit | 38.7 | % | Thalassaemia |
|  |  |  | RBC | 6.01 | 10^6^/μL | Thalassaemia |
|  |  | Day 7/EOT | Hemoglobin | 11.7 | g/ dL | Thalassaemia |
|  |  |  | Hematocrit | 39.0 | % | Thalassaemia |
|  |  |  | RBC | 6.15 | 10^6^/μL | Thalassaemia |
|  |  | Day 9 | Hemoglobin | 11.6 | g/ dL | Thalassaemia |
|  |  |  | Hematocrit | 39.2 | % | Thalassaemia |
|  |  |  | RBC | 6.06 | 10^6^/μL | Thalassaemia |
|  |  | Day 14 | Hemoglobin | 11.6 | g/ dL | Thalassaemia |
|  |  |  | RBC | 6.12 | 10^6^/μL | Thalassaemia |
|  |  | Day 28 | Hemoglobin | 11.6 | g/ dL | Thalassaemia |
|  |  |  | RBC | 6.00 | 10^6^/μL | Thalassaemia |
|  |  | Day 60 | Hemoglobin | 12.3 | g/ dL | Thalassaemia |
|  |  |  | RBC | 6.37 | 10^6^/μL | Thalassaemia |
|  | 007-S065 (male) | Day 4 | WBC | 2.70 | 10^3^/μL | AE: WBC count decreased |
|  |  |  | Absolute neutrophil counts | 1.0098 |  | AE: Neutrophil count decreased |
|  |  | Day 7/EOT | WBC | 4.28 | 10^3^/μL | AE: WBC count decreased |
|  |  |  | Absolute neutrophil counts | 1.74624 | - | AE: Neutrophil count decreased |
|  | 007-S133 (female) | Day 2 | WBC | 3.92 | 10^3^/μL | Leukopenia |
|  |  | Day 4 | WBC | 2.57 | 10^3^/μL | Leukopenia |
|  |  | Day 7/EOT | WBC | 4.12 | 10^3^/μL | Leukopenia |
|  |  | Day 9 | WBC | 4.27 | 10^3^/μL | Leukopenia |
|  |  | Day 14 | WBC | 3.53 | 10^3^/μL | Leukopenia |
|  |  | Day 28 | WBC | 3.03 | 10^3^/μL | Leukopenia |
|  | 007-S156 (female) | Day 4 | WBC | 2.32 | 10^3^/μL | COVID-19 related |
|  |  |  | Neutrophils | 32.7 | % | COVID-19 related |
|  |  |  | Lymphocytes | 51 | % | COVID-19 related |
|  |  |  | Monocytes | 15.1 | % | COVID-19 related |
|  | 001-S157 (female) | Day 4 | WBC | 3.09 | 10^3^/μL | AE: WBC count decreased |
|  |  |  | Neutrophils | 33.0 | % | AE: Neutrophil count decreased |
| Abbreviation: AE, adverse event; EOT, end of treatment; RBC, red blood cell; WBC, white blood cell | | | | | | |

### 1.3.2. Biochemistry

**Table 5** summarizes CS abnormalities in biochemistry.

Most CS abnormalities in participants were linked to underlying diseases or COVID-19. Notably, there was a trend of increasing CPK levels across the three groups. Elevation of CPK values was observed in eight participants of the two SNS812 treatment groups. All the CPK elevations were transient, resolved rapidly, showed no temporal relationship with SNS812, and occurred following vigorous physical activity undertaken by patients after COVID-19 symptom recovery. Participants S013 and S140 in the 100-mg group and S003, S010, S046, S086, S119, and S152 in the 200-mg group experienced increased CPK. Participant S016 in the 100-mg group showed an increase in AST and ALT on Day 2, which were classified as Grade 1, resolved, and considered unlikely related to the IP. Participant S140 in the 100-mg group also experienced an increase in AST and ALT. Additionally, participant S152 in the 200-mg group had an increase in AST levels on Day 14, which the investigator attributed to exercise-induced myositis, as this event was not observed in subsequent visits.

Other participants in the 100-mg and 200-mg groups did not exhibit CS abnormalities during the study period, and no identifiable trends related to the IP were noted in biochemistry.

- **Table 5: Abnormality with Clinical Significance in Biochemistry**

| **Group** | **Participant** | **Visit** | **Item** | **Value** | **Unit** | **Reason** |
| --- | --- | --- | --- | --- | --- | --- |
| **SNS812**  **200 mg** | 001-S003 (male) | Screening | Creatine Kinase | 882 | U/L | COVID-19 related |
|  |  | Day 2 | Creatine Kinase | 238 | U/L | COVID-19 related |
|  | 001-S010 (male) | Day 4 | Creatine Kinase | 1881 | U/L | AE: Blood creatine phosphokinase increased |
|  |  | Day 7/EOT | Creatine Kinase | 413 | U/L | AE: Blood creatine phosphokinase increased |
|  |  | Day 9 | Creatine Kinase | 232 | U/L | AE: Blood creatine phosphokinase increased |
|  |  | Day 14 | Creatine Kinase | 504 | U/L | AE: Blood creatine phosphokinase increased |
|  |  | Day 28 | Creatine Kinase | 501 | U/L | AE: Blood creatine phosphokinase increased |
|  |  | Day 60 | Creatine Kinase | 847 | U/L | AE: Blood creatine phosphokinase increased |
|  | 007-S046 (female) | Day 14 | Creatine Kinase | 1580 | U/L | AE: Blood creatine phosphokinase increased |
|  | 001-S061 (female) | Screening | Triglyceride | 209 | mg/dL | Hyperlipidaemia |
|  |  | Day 2 | Triglyceride | 383 | mg/dL | Hyperlipidaemia |
|  |  | Day 4 | Triglyceride | 295 | mg/dL | Hyperlipidaemia |
|  |  | Day 7/EOT | Triglyceride | 368 | mg/dL | Hyperlipidaemia |
|  |  | Day 9 | Triglyceride | 335 | mg/dL | Hyperlipidaemia |
|  |  | Day 14 | Triglyceride | 243 | mg/dL | Hyperlipidaemia |
|  |  | Day 60 | Triglyceride | 238 | mg/dL | Hyperlipidaemia |
|  | 001-S081 (male) | Day 2 | Serum Creatinine | 1.56 | mg/dL | AE: Blood creatinine increased |
|  |  | Day 4 | Serum Creatinine | 1.41 | mg/dL | AE: Blood creatinine increased |
|  |  | Day 7/EOT | Serum Creatinine | 1.48 | mg/dL | AE: Blood creatinine increased |
|  | 006-S086 (male) | Day 14 | Creatine Kinase | 1195 | U/L | AE: Blood creatine phosphokinase increased |
|  | 007-S117 (male) | Day 9 | Total Bilirubin | 1.91 | mg/dL | AE: Blood bilirubin increased |
|  |  | Day 14 | Total Bilirubin | 2.06 | mg/dL | AE: Blood bilirubin increased |
|  | 007-S152 (female) | Day 14 | Aspartate Aminotransferase | 114 | U/L | AE: Myositis |
|  |  |  | Creatine Kinase | 4889 | U/L | AE: Myositis |
| **SNS812**  **100 mg** | 001-S001 (male) | Screening | Triglyceride | 255 | mg/dL | Hyperlipidaemia |
|  |  |  | Total Cholesterol | 230 | mg/dL | Hyperlipidaemia |
|  |  | Day 2 | Glucose, Random | 143 | mg/dL | Diabetes mellitus |
|  |  |  | Total Cholesterol | 238 | mg/dL | Hyperlipidaemia |
|  |  | Day 4 | Glucose, Random | 177 | mg/dL | Diabetes mellitus |
|  |  |  | Triglyceride | 222 | mg/dL | Hyperlipidaemia |
|  |  |  | Total Cholesterol | 223 | mg/dL | Hyperlipidaemia |
|  |  | Day 7/EOT | Glucose, Random | 147 | mg/dL | Diabetes mellitus |
|  |  |  | Triglyceride | 178 | mg/dL | Hyperlipidaemia |
|  |  |  | Total Cholesterol | 229 | mg/dL | Hyperlipidaemia |
|  |  | Day 9 | Glucose, Random | 170 | mg/dL | Diabetes mellitus |
|  |  |  | Triglyceride | 167 | mg/dL | Hyperlipidaemia |
|  |  |  | Total Cholesterol | 243 | mg/dL | Hyperlipidaemia |
|  |  | Day 14 | Triglyceride | 217 | mg/dL | Hyperlipidaemia |
|  |  |  | Total Cholesterol | 264 | mg/dL | Hyperlipidaemia |
|  |  | Day 28 | Triglyceride | 185 | mg/dL | Hyperlipidaemia |
|  |  |  | Total Cholesterol | 269 | mg/dL | Hyperlipidaemia |
|  |  | Day 60 | Glucose, Random | 132 | mg/dL | Diabetes mellitus |
|  |  |  | Triglyceride | 285 | mg/dL | Hyperlipidaemia |
|  |  |  | Total Cholesterol | 271 | mg/dL | Hyperlipidaemia |
|  | 001-S009 (female) | Screening | Triglyceride | 247 | mg/dL | Hypertriglyceridaemia |
|  |  | Day 2 | Triglyceride | 174 | mg/dL | Hypertriglyceridaemia |
|  |  | Day 4 | Triglyceride | 227 | mg/dL | Hypertriglyceridaemia |
|  |  | Day 7/EOT | Triglyceride | 166 | mg/dL | Hypertriglyceridaemia |
|  |  | Day 9 | Triglyceride | 158 | mg/dL | Hypertriglyceridaemia |
|  |  | Day 14 | Triglyceride | 168 | mg/dL | Hypertriglyceridaemia |
|  |  | Day 28 | Triglyceride | 179 | mg/dL | Hypertriglyceridaemia |
|  |  | Day 60 | Triglyceride | 189 | mg/dL | Hypertriglyceridaemia |
|  | 001-S013 (male) | Screening | Total Cholesterol | 237 | mg/dL | Blood cholesterol increased |
|  |  | Day 2 | Total Cholesterol | 216 | mg/dL | Blood cholesterol increased |
|  |  | Day 4 | Total Cholesterol | 203 | mg/dL | Blood cholesterol increased |
|  |  | Day 7/EOT | Creatine Kinase | 1026 | U/L | AE: Blood creatine phosphokinase increased |
|  |  |  | Total Cholesterol | 210 | mg/dL | Blood cholesterol increased |
|  |  | Day 9 | Creatine Kinase | 543 | U/L | AE: Blood creatine phosphokinase increased |
|  |  |  | Total Cholesterol | 214 | mg/dL | Blood cholesterol increased |
|  |  | Day 14 | Creatine Kinase | 363 | U/L | AE: Blood creatine phosphokinase increased |
|  |  |  | Total Cholesterol | 214 | mg/dL | Blood cholesterol increased |
|  |  | Day 28 | Creatine Kinase | 498 | U/L | AE: Blood creatine phosphokinase increased |
|  |  |  | Total Cholesterol | 216 | mg/dL | Blood cholesterol increased |
|  |  | Day 60 | Creatine Kinase | 599 | U/L | AE: Blood creatine phosphokinase increased |
|  |  |  | Total Cholesterol | 223 | mg/dL | Blood cholesterol increased |
|  | 001-S016 (male) | Day 2 | Aspartate Aminotransferase | 73 | U/L | AE: Aspartate aminotransferase increased |
|  |  |  | Alanine Aminotransferase | 119 | U/L | AE: Alanine aminotransferase increased |
|  | 001-S024 (female) | Screening | Total Cholesterol | 273 | mg/dL | Hyperlipidaemia |
|  |  | Day 2 | Total Cholesterol | 249 | mg/dL | Hyperlipidaemia |
|  |  | Day 4 | Total Cholesterol | 249 | mg/dL | Hyperlipidaemia |
|  |  | Day 7/EOT | Total Cholesterol | 205 | mg/dL | Hyperlipidaemia |
|  |  | Day 9 | Triglyceride | 236 | mg/dL | Hyperlipidaemia |
|  |  |  | Total Cholesterol | 205 | mg/dL | Hyperlipidaemia |
|  |  | Day 14 | Total Cholesterol | 230 | mg/dL | Hyperlipidaemia |
|  |  | Day 28 | Total Cholesterol | 258 | mg/dL | Hyperlipidaemia |
|  | 006-S140 (female) | Day 2 | Alanine Aminotransferase | 211 | U/L | AE: Alanine aminotransferase increased |
|  |  | Day 7/EOT | Alanine Aminotransferase | 288 | U/L | AE: Alanine aminotransferase increased |
|  |  |  | Aspartate Aminotransferase | 200 | U/L | AE: Aspartate aminotransferase increased |
|  |  | Day 9 | Aspartate Aminotransferase | 231 | U/L | AE: Aspartate aminotransferase increased |
|  |  | Day 14 | Alanine Aminotransferase | 655 | U/L | AE: Alanine aminotransferase increased |
|  |  |  | Aspartate Aminotransferase | 394 | U/L | AE: Aspartate aminotransferase increased |
|  |  | Day 28 | Creatine Kinase | 575 | U/L | AE: Blood creatine phosphokinase increased |
|  | 001-S060 (male) | Screening | Triglyceride | 443 | mg/dL | Hypertriglyceridaemia |
|  |  | Day 2 | Triglyceride | 494 | mg/dL | Hypertriglyceridaemia |
|  |  | Day 4 | Triglyceride | 609 | mg/dL | Hypertriglyceridaemia |
|  |  | Day 7/EOT | Triglyceride | 558 | mg/dL | Hypertriglyceridaemia |
|  |  | Day 9 | Triglyceride | 445 | mg/dL | Hypertriglyceridaemia |
|  |  | Day 28 | Triglyceride | 311 | mg/dL | Hypertriglyceridaemia |
|  |  | Day 60 | Triglyceride | 532 | mg/dL | Hypertriglyceridaemia |
|  | 007-S153 (female) | Screening | Total Cholesterol | 243 | mg/dL | Hyperlipidaemia |
|  |  |  | Triglyceride | 218 | mg/dL | Hyperlipidaemia |
|  |  | Day 2 | Total Cholesterol | 250 | mg/dL | Hyperlipidaemia |
|  |  | Day 4 | Total Cholesterol | 244 | mg/dL | Hyperlipidaemia |
|  |  |  | Triglyceride | 210 | mg/dL | Hyperlipidaemia |
|  |  | Day 7/EOT | Total Cholesterol | 255 | mg/dL | Hyperlipidaemia |
|  |  |  | Triglyceride | 277 | mg/dL | Hyperlipidaemia |
|  |  | Day 9 | Total Cholesterol | 248 | mg/dL | Hyperlipidaemia |
|  |  |  | Triglyceride | 464 | mg/dL | Hyperlipidaemia |
|  |  | Day 14 | Total Cholesterol | 254 | mg/dL | Hyperlipidaemia |
|  |  |  | Triglyceride | 216 | mg/dL | Hyperlipidaemia |
|  |  | Day 28 | Total Cholesterol | 241 | mg/dL | Hyperlipidaemia |
|  |  |  | Triglyceride | 295 | mg/dL | Hyperlipidaemia |
|  |  | Day 60 | Total Cholesterol | 217 | mg/dL | Hyperlipidaemia |
| **Placebo** | 001-S027 (female) | Day 14 | Total Bilirubin | 1.44 | mg/dL | AE: Blood bilirubin increased |
|  |  | Day 28 | Total Bilirubin | 1.03 | mg/dL | AE: Blood bilirubin increased |
|  | 001-S030 (male) | Day 60 | Serum Creatinine | 1.43 | mg/dL | AE: Blood creatinine increased |
|  | 001-S064 (female) | Day 2 | Bicarbonate | 29.1 | mmol/L | AE: Dysgeusia |
|  | 007-S133 (female) | Screening | Total Cholesterol | 242 | mg/dL | Hyperlipidaemia |
|  |  | Day 2 | Total Cholesterol | 217 | mg/dL | Hyperlipidaemia |
|  |  | Day 4 | Total Cholesterol | 226 | mg/dL | Hyperlipidaemia |
|  |  | Day 7/EOT | Total Cholesterol | 214 | mg/dL | Hyperlipidaemia |
|  |  | Day 9 | Total Cholesterol | 235 | mg/dL | Hyperlipidaemia |
|  |  | Day 14 | Total Cholesterol | 236 | mg/dL | Hyperlipidaemia |
|  |  | Day 28 | Total Cholesterol | 238 | mg/dL | Hyperlipidaemia |
| Abbreviation: AE, adverse event; EOT, end of treatment | | | | | | |

### 1.3.3.  Coagulation Indices

No participants in the 100-mg and 200-mg groups exhibited CS abnormalities during the study period, and no identifiable trends of abnormalities in coagulation indices were found.

### 1.3.4.  Urinalysis

CS abnormalities in urinalysis are shown in **Table 6**.

All CS abnormalities observed in participants were attributed to underlying diseases or menstruation.

Other participants in the 100-mg and 200-mg groups did not show CS abnormalities during the study period, and no identifiable trends related to the IP were noted in urinalysis.

- **Table 6: Abnormality with Clinical Significance in Urinalysis**

| **Group** | **Participant** | **Visit** | **Item** | **Value** | **Unit** | **Reason** |
| --- | --- | --- | --- | --- | --- | --- |
| **SNS812**  **100 mg** | 001-S001 (male) | Screening | Protein | 30 | mg/dL | Diabetes mellitus |
|  |  | Day 2 | Protein | 100 | mg/dL | Diabetes mellitus |
|  |  | Day 4 | Protein | 30 | mg/dL | Diabetes mellitus |
|  |  | Day 7/EOT | Protein | 15 | mg/dL | Diabetes mellitus |
|  | 001-S009 (female) | Day 4  Day 7/EOT | Occult Blood | 3+ | N/A | Urinary tract infection |
|  |  |  | Occult Blood | 3+ | N/A | Urinary tract infection |
| **SNS812**  **200 mg** | 001-S020 (male) | Screening | Occult Blood | 2+ | N/A | C3 glomerulopathy |
|  |  |  | Protein | 15 | mg/dL | C3 glomerulopathy |
|  |  | Day 2 | Occult Blood | 2+ | N/A | C3 glomerulopathy |
|  |  |  | Protein | 15 | mg/dL | C3 glomerulopathy |
|  |  | Day 4 | Occult Blood | 2+ | N/A | C3 glomerulopathy |
|  |  | Day 7/EOT | Occult Blood | +/- | N/A | C3 glomerulopathy |
|  | 007-S152 (female) | Day 28 | Occult Blood | 2+ | N/A | Menstruation |
| **Placebo** | NA |  |  |  |  |  |
| Abbreviation: EOT, end of treatment; NA, not applicable; RBC, red blood cell; WBC, white blood cell | | | | | | |

## 1.4.  Vital Signs, Physical Findings, and Other Observations Related to Safety

### 1.4.1. Vital Signs

Generally, no clinically significant trends were observed.

### 1.4.2.  12-Lead ECG

Overall, no clinically significant trends were noted.

### 1.4.3. Chest X-Ray

Generally, no clinically significant trends were observed.

### 1.4.4.  SpO_2_ and the Respiratory Support Status

Overall, no clinically significant trends were observed.

## 1.5.  Safety Conclusions

Safety data were reported for the SS population, which included all randomized participants who received at least one dose of the study drug. Out of 134 participants, 72 (53.73%) experienced 158 TEAEs, no participants reported TRAEs, and 1 participant had SAEs. The majority of TEAEs (111 [70.25%]) were classified as mild in severity. The investigator determined that all TEAEs were either unrelated (136 [86.08%]) or unlikely related (22 [13.92%]) to the study treatment, and no actions were taken regarding the study treatment; 138 (87.34%) TEAEs had resolved. Most TEAEs were associated with participants’ underlying conditions or the nature of COVID-19, with the most common being anosmia (20 [14.93%]), dysgeusia (12 [8.96%]), and diarrhea (11 [8.21%]). The two SAEs (increased ALT and increased AST) were related to the underlying condition of participant S140. No CS safety trends were identified during the evaluations. In summary, SNS812 was generally safe and well tolerated among participants with mild-to-moderate COVID-19.
